# Generalizing the Balance Heuristic Estimator in Multiple Importance Sampling

**DOI:** 10.3390/e24020191

**Published:** 2022-01-27

**Authors:** Mateu Sbert, Víctor Elvira

**Affiliations:** 1 Informatics and Applications Institute, Girona University, 17003 Girona, Spain; 2 School of Mathematics, University of Edinburgh, Edinburgh EH8 9YL, UK; 3 The Alan Turing Institute, London NW1 2DB, UK

**Keywords:** Monte Carlo, importance sampling, balance heuristic, variance reduction, chi-square divergence, Kullback–Leibler divergence, cross entropy

## Abstract

In this paper, we propose a novel and generic family of multiple importance sampling estimators. We first revisit the celebrated balance heuristic estimator, a widely used Monte Carlo technique for the approximation of intractable integrals. Then, we establish a generalized framework for the combination of samples simulated from multiple proposals. Our approach is based on considering as free parameters both the sampling rates and the combination coefficients, which are the same in the balance heuristics estimator. Thus our novel framework contains the balance heuristic as a particular case. We study the optimal choice of the free parameters in such a way that the variance of the resulting estimator is minimized. A theoretical variance study shows the optimal solution is always better than the balance heuristic estimator (except in degenerate cases where both are the same). We also give sufficient conditions on the parameter values for the new generalized estimator to be better than the balance heuristic estimator, and one necessary and sufficient condition related to $\chi^{2}$ divergence. Using five numerical examples, we first show the gap in the efficiency of both new and classical balance heuristic estimators, for equal sampling and for several state of the art sampling rates. Then, for these five examples, we find the variances for some notable selection of parameters showing that, for the important case of equal count of samples, our new estimator with an optimal selection of parameters outperforms the classical balance heuristic. Finally, new heuristics are introduced that exploit the theoretical findings.

## 1. Introduction

Multiple importance sampling (MIS) is a Monte Carlo technique widely used in the literature of signal processing, computational statistics, and computer graphics for approximating complicated integrals. In its basic configuration, it works by drawing random samples from several proposal distributions (also called techniques) and weighting them appropriately in such a way that an estimator built with the pairs of weighted samples is consistent. Since the publication of [1], the celebrated *balance heuristic* estimator has been extensively used in the Monte Carlo literature, with an unprecedented success in the computer graphics industry (Eric Veach has been awarded with several prizes because of his contributions in the MIS literature, where the balance heuristic is arguably the most relevant one). In the balance heuristic method, different samples are simulated from each proposal and the traditional IS weight is assigned to each of them. Unlike the standard IS estimator, all the weighted samples are combined with an extra weighting, in such a way the resulting estimator typically shows a reduced variance. Its superiority in terms of variance with regard to other traditional combination schemes has been recently shown in [2], where a framework is established for sampling and weighting in MIS under equal number of samples per technique. The balance heuristic, also called the *deterministic mixture* [3], has been widely used in the literature of MIS. Further efficient variance reduction techniques are proposed in [4,5,6] also in the context of MIS, still with equal counts from each technique. Provably better estimators [7] and heuristically better ones [8,9] have been presented that use a different count of samples than equal count for all techniques. In [10], it has been shown the relationship of a better count of samples with generalized weighted means. The balance heuristic is also present in most of successful adaptive IS (AIS) methods, see [11,12,13,14,15], in particular in the case where all techniques are used to simulate the same number of samples. Recently, MIS has returned to the main focus in computer graphics in [16], where it is shown that allowing weights to be negative, the reduction over balance heuristics of the resulting MIS estimator can be higher than the one predicted by Veach bounds [17]; in [18], where one of the sampling techniques is optimized to decrease the overall variance of the resulting MIS estimator; in [19], where the weights are made proportional to the quotients of second moments divided by the variances of the independent techniques; in [20], where MIS is generalized to uncountably infinite sets of techniques.

Interestingly, the balance heuristic has two properties in the assigned weights. First, all techniques appear at the denominator of the weight of a specific technique. Second, they appear in a form of a mixture, with coefficients proportional to the number of samples simulated from each technique. In this paper, we relax this constraint providing a generalized weighting/combining family of estimators that has the balance heuristic as a particular case. First, we show that it is possible to use a specific set of coefficients to decide the amount of samples per technique, and a different set of coefficients to be applied as the importance weight. Second, we study four different cases fixing some of these coefficients (sampling and/or weighting), and we give the optimal solution for the rest of coefficients in such a way the variance of the MIS estimator is minimized. Note that, the novel estimator always outperforms the balance heuristic under the optimal choice of those coefficients. In five numerical examples we show that, under an adequate choice of parameters, the novel estimator outperforms the celebrated balance heuristic.

The rest of the paper is structured as follows. Section 2 revisits the balance heuristic estimator. In Section 3, we propose the new family of estimators that generalizes the balance heuristic. We address five cases of special interest, depending on which parameters are free and the number of samples simulated from each technique. We then, in Section 4, discuss the different singular points in the variances of the estimators considered. In Section 5, we give a necessary and sufficient condition, in terms of $\chi^{2}$ divergence (and its approximation as a Kullback–Leibler divergence), for the variance of the new estimator to be better than the one of balance heuristics. Finally, we conclude the paper with five numerical examples in Section 6, propose some heuristics, and give some conclusions in Section 7.

## 2. Balance Heuristic Estimator

The goal in IS is usually the estimation of the value of integral μ=∫f(x)dx. In MIS, $n_{i}$ samples, ${\left\{X_{i,j}\right\}}_{j=1}^{n_{i}}$, are simulated from a set of available probability density functions (pdfs), ${\left\{p_{i}\right\}}_{i=1}^{n}$. The MIS estimator introduced by Veach and Guibas [1] is given by

(1) $$Z=\sum_{i=1}^{n}\frac{1}{n_{i}}\sum_{j=1}^{n_{i}}w_{i}\left(X_{i,j}\right)\frac{f(X_{i,j})}{p_{i}\left(X_{i,j}\right)},$$

where $w_{i}\left(x\right)$ is a weight function associated to the *i*-th proposal that fulfills both following conditions. First, the weights must sum up to one in all points of the domain where the value of the function is different from zero, i.e., $\sum_{i=1}^{n}w_{i}\left(x\right)=1$, ∀x where f(x)≠0, Second, for all *x* where $p_{i}\left(x\right)=0$, then $w_{i}\left(x\right)=0$. In this paper, we consider only weighting functions that yield *Z* unbiased.

The *balance heuristic* estimator is a particular case of Equation (1) where the weight function is given by

(2) $$w_{i}\left(x\right)=\frac{n_{i}p_{i}\left(x\right)}{\sum_{k=1}^{n}n_{k}p_{k}\left(x\right)},$$

which can be written too as

(3) $$w_{i}\left(x\right)=\frac{\alpha_{i}p_{i}\left(x\right)}{\sum_{k=1}^{n}\alpha_{k}p_{k}\left(x\right)},$$

where $n_{i}=\alpha_{i}N$. In this case, the estimator in Equation (1) becomes the balance heuristic or deterministic mixture estimator given by

(4) $$\begin{array}{ccc} F & = & \sum_{i=1}^{n}\frac{1}{n_{i}}\sum_{j=1}^{n_{i}}\frac{\alpha_{i}f\left(X_{i,j}\right)}{\sum_{k=1}^{n}\alpha_{k}p_{k}\left(X_{i,j}\right)} \end{array}$$

(5) $$\begin{array}{ccc} & = & \frac{1}{N}\sum_{i=1}^{n}\sum_{j=1}^{n_{i}}\frac{f(X_{i,j})}{\sum_{k=1}^{n}\alpha_{k}p_{k}\left(X_{i,j}\right)}. \end{array}$$

### 2.1. Interpretation of F and General Notation of the Paper

Note that *F* is also called *deterministic mixture scheme* [3] or *multi-sample MISestimator* [7,17], as opposed to the case where all independent and identically distributed (i.i.d.) samples are from the mixture $\psi_{\alpha }=\sum_{k=1}^{n}\alpha_{k}p_{k}\left(x\right)$. The latter alternative is called the *randomized balance heuristic* or *one-sample MIS estimator*, and the estimator is denoted by F. This alternative is a subtle variation of *F* that simply modifies the sampling by simulating the *N* total samples i.i.d. from the mixture $\psi_{\alpha }$ (instead of deterministically choosing the number of samples per proposal). Then, the estimator is the exact same expression of Equation (4). This randomized version F allows also for a re-interpretation of the deterministic balance heuristic, *F*, that can be seen as a mixture sampling (note that the mixture is present in the denominator of all importance weights) with variance reduction using stratified sampling (see Appendix 1 of [2] for a detailed discussion). In the randomized version, $n_{i}$ is then a random variable with expected value $\alpha_{i}N$, for all *i*. In summary, in this paper we refer as *deterministic mixture estimator* the cases where the number of samples per technique is deterministic, e.g., *F*, and *random mixture estimator* when there is i.i.d. sampling from the mixture (and hence $n_{i}$ is a random variable), e.g., F. We represent the randomized estimators with calligraphic letters. All estimators, unless the opposite is clearly stated, are deterministic mixture estimators.

Finally, we denote estimators with the superindex${}^{1}$ when they are versions of a specific estimator but with a number of samples normalized to 1, e.g., $F^{1}$ is the normalized version of *F*. In other words, even if the estimators require that all the numbers of samples per technique are $n_{i}\in R$, we use these normalized estimators to denote the variance normalized to 1 sample, which simplifies the comparison across estimators (for *N* total samples, the variance of the estimator would be just the variance of $F^{1}$ divided by *N*).

In Table 1 we show the naming convention used in this paper.

### 2.2. Rationale

In Theorems 9.2 and 9.4 of [17], the variances of $Z^{1}$ and its randomized version $Z^{1}$ are given, i.e., the version where instead of deterministically selecting $n_{i}$, all the samples are directly simulated from $\sum_{k=1}^{n}\alpha_{k}p_{k}\left(x\right)$. By subtracting their values we have

(6) $$\begin{array}{c} V\left[Z^{1}\right]-V\left[Z^{1}\right]=\sum_{i}\alpha_{i}{\mu^{\prime }}_{i}^{2}-\mu^{2}, \end{array}$$

where (we use here a notation to be consistent with notation in [7])

(7) $$\begin{array}{c} {\mu^{\prime }}_{i}=\frac{1}{\alpha_{i}}\int w_{i}\left(x\right)f\left(x\right)dx, \end{array}$$

and thus

(8) $$\begin{array}{c} \sum_{i}\alpha_{i}{\mu^{\prime }}_{i}=\mu . \end{array}$$

This result generalizes several particular cases derived in [7] and [2], in the context of a variance analysis of MIS estimators. From Equation (6) (see Appendix A) we have that

(9) $$\begin{array}{c} V\left[Z^{1}\right]\leq V\left[Z^{1}\right], \end{array}$$

and equality only happens (apart from the case when both variances $V\left[Z^{1}\right],V\left[Z^{1}\right]$ are zero) when for all *i* all $\mu_{i}^{\prime }$ are equal. One example is given by taking in Equation (7) for all *i*, $w_{i}\left(x\right)=w_{i}$ constant and $\alpha_{i}=w_{i}$. We also show in Appendix A that, for the particular case when $\alpha_{i}=1/n$ and f(x)≥0, the upper bound for improvement of deterministic versus non-deterministic estimator is given by

(10) $$\begin{array}{c} V\left[Z^{1}\right]-V\left[Z^{1}\right]\leq \left(n-1\right)\mu^{2}, \end{array}$$

where the bound would be approached when there is an index *k* such that for all i≠k, $\mu_{k}^{\prime }>>\mu_{i}^{\prime }$. In Theorem 9.4 of [17], it is shown that the optimal weighting functions $w_{i}\left(x\right)$ for Z (i.e., those that minimize their variance V[Z]), are the balance heuristic weights, Equation (3); therefore, the optimal case is Z≡F, where F is the random mixture estimator. This is, for any estimator Z, when using the same distribution of samples, also taking into account Equation (9) (see also [7]), it always holds that

(11) $$\begin{array}{c} V[F]\leq V[F]\leq V[Z]. \end{array}$$

Further, in Theorem 9.2 of [17], it is proved that the estimator that optimizes the second moment of $Z^{1}$ estimator, this is, $V\left[Z^{1}\right]+\sum_{i}\alpha_{i}{\mu^{\prime }}_{i}^{2}$, is the balance heuristic estimator. Thus, it seems clear that for improvement we have to look for a deterministic estimator, which should be a generalization of balance heuristic mixture estimator *F*. This is presented in the next section.

### 2.3. Realistic Applications

#### 2.3.1. Global Illumination

The rendering equation [21] tells us that the radiance $L_{out}\left(x,\omega_{out}\right)$ exiting point *x* in direction $\omega_{out}$ is given by

(12) $$L_{out}\left(x,\omega_{out}\right)=L_{e}\left(x,\omega_{out}\right)+\int_{\Omega }L_{in}\left(x,\omega_{in}\right)\rho \left(x,\omega_{in},\omega_{out}\right)\cos\theta d\omega_{in},$$

where $L_{e}\left(x,\omega_{out}\right)$ is the emitted radiance, $L_{in}\left(x,\omega_{in}\right)$ is the incoming radiance at *x* from direction $\omega_{in}$, with the integral extended to the hemisphere Ω above *x*, θ is the angle of the normal at *x* with $\omega_{in}$, and ρ is the bidirectional reflectance distribution function, which gives the fraction of luminance incoming from $\omega_{in}$ and scattered in the direction $\omega_{out}$. To approximate the integral in (12), multiple importance sampling is applied with pdfs that sample, respectively, the incoming radiance and the bidirectional reflectance.

#### 2.3.2. Bayesian Inference

Multiple importance sampling (MIS) is often applied in Bayesian inference [22]. In this context, the posterior distribution is usually intractable, and computing its moments analytically is impossible; therefore, approximate methods are required. MIS is particularly well suited for cases when the posterior distribution is multimodal or needs to be approximated by a mixture of densities [2].

Typically, a set of observations, $y\in R^{d_{y}}$, are available. The inference task consists in estimating probabilistically some hidden parameters and/or latent variables $x\in R^{d_{y}}$, which are connected to the observations through a statistical model. The relation between observations and unknown parameters is encoded in the likelihood, ℓ(y|x), and the prior knowledge on x is described through the prior distribution $p_{0}\left(x\right)$. Through the Bayes’ rule, we can obtain the posterior distribution of the unknowns as

(13) $$\tilde{\pi }\left(x|y\right)=\frac{\ell \left(y|x\right)p_{0}\left(x\right)}{Z(y)},$$

where $Z\left(y\right)=\int \ell \left(y|x\right)p_{0}\left(x\right)dx$ is the marginal likelihood.

In this scenario, we can be interested in computing a moment h(x) of the posterior, which is an expectation (hence an integral) of the following form:

(14) $$\mu =E_{\tilde{\pi }}\left[h\left(x\right)\right]=\int h\left(x\right)\tilde{\pi }\left(x|y\right)dx,$$

where $h:R^{d_{x}}\to R$. In connection with the notation of this paper, $f\left(x\right)=h\left(x\right)\tilde{\pi }\left(x|y\right)$. In the context of Bayesian inference, there are several ways to select the techniques, ${\left\{p_{i}\left(x\right)\right\}}_{i=1}^{n}$. For instance, they can be deterministically set a priori, e.g., as Laplace approximations at different points [23]. Alternatively, they can be adapted over iterations via iterative stochastic mechanisms [24]. The latter technique is called adaptive importance sampling (AIS) and its literature is vast (see [15] for a recent review).

## 3. Generalized Multiple Importance Sampling Balance Heuristic Estimator

In classic balance heuristic, both the number of samples ${\left\{n_{i}=\alpha_{i}N\right\}}_{i=1}^{n}$ and the weights of the mixture of pdfs, ${\left\{\alpha_{i}\right\}}_{i=1}^{n}$, are given by the same set of parameters. Let us consider now the estimator of Equation (4), where we relax the dependence between the number of samples $n_{i}$, and the associated coefficient $\alpha_{i}$, i.e., now $n_{i}=\beta_{i}N$, $\beta_{i}>0$, $\sum_{i=1}^{n}\beta_{i}=1$, where in general $\alpha_{i}\neq \beta_{i}$ (otherwise, we recover *F*). We now define the estimator

(15) $$\begin{array}{ccc} G & = & \sum_{i=1}^{n}\frac{\alpha_{i}}{n_{i}}\sum_{j=1}^{n_{i}}\frac{f(X_{i,j})}{\sum_{k=1}^{n}\alpha_{k}p_{k}\left(X_{i,j}\right)} \end{array}$$

(16) $$\begin{array}{ccc} & = & \frac{1}{N}\sum_{i=1}^{n}\frac{\alpha_{i}}{\beta_{i}}\sum_{j=1}^{n_{i}}\frac{f(X_{i,j})}{\sum_{k=1}^{n}\alpha_{k}p_{k}\left(X_{i,j}\right)}. \end{array}$$

Note that *G* is a particular case of *Z*, with weights $w_{i}=\frac{\alpha_{i}p_{i}\left(x\right)}{\sum_{k=1}^{n}\alpha_{k}p_{k}\left(X_{i,j}\right)}$ in Equation (1). Note also that the balance heuristic *F* is a particular case of *G*, i.e., in general we do not impose the restriction of $\alpha_{i}=\frac{n_{i}}{N}$. Although the allocation of samples will be different for *F* and *G*, we remark that both estimators require the same number of simulations and evaluations. Finally, we note that a close examination of (16) allows us to re-interpret the importance weight associated to each sample. The mixture parametrized with ${\left\{n_{i}=\alpha_{i}N\right\}}_{i=1}^{n}$ appears in its denominator, but since this mixture does not reflect the sampling procedure (the true mixture proposal in sampling is the one parametrized by ${\left\{n_{i}=\beta_{i}N\right\}}_{i=1}^{n}$), the importance weight of a sample simulated from the technique $\beta_{i}$, is corrected by the factor $\alpha_{i}/\beta_{i}$ in order to account for this mismatch.

Estimator *G* can be rewritten as $G=\sum_{i=1}^{n}\alpha_{i}G_{i}$, where

(17) $$\begin{array}{ccc} G_{i} & = & \frac{1}{n_{i}}\sum_{j=1}^{n_{i}}\frac{f(X_{i,j}))}{\sum_{k=1}^{n}\alpha_{k}p_{k}\left(X_{i,j}\right))}. \end{array}$$

Note also that *G* depends of two sets of parameters, ${\left\{\alpha_{i}\right\}}_{i=1}^{n}\;and\;{\left\{\beta_{i}\right\}}_{i=1}^{n}$. In the particular case where $\beta_{i}=\alpha_{i},\forall i$, the estimator *G* becomes *F*. Let us consider the case with $n_{i}=1$. Then,

(18) $$\begin{array}{ccc} G_{i}^{\prime } & = & \frac{f(x)}{\sum_{k=1}^{n}\alpha_{k}p_{k}\left(x\right)}, \end{array}$$

with expectation

(19) $$\begin{array}{c} E\left[G_{i}^{\prime }\right]=\int \frac{f\left(x\right)p_{i}\left(x\right)}{\sum_{k=1}^{n}\alpha_{k}p_{k}\left(x\right)}dx\equiv \mu_{i}^{\prime }, \end{array}$$

and variance

(20) $$\begin{array}{ccc} {\sigma^{\prime }}_{i}^{2} & = & \int \frac{f^{2}\left(x\right)p_{i}\left(x\right)}{{\left(\sum_{k=1}^{n}\alpha_{k}p_{k}\left(x\right)\right)}^{2}}dx-{\left(\mu_{i}^{\prime }\right)}^{2}. \end{array}$$

Observe that $E\left[G_{i}\right]=E\left[G_{i}^{\prime }\right]=\mu_{i}^{\prime }$, and $V\left[G_{i}\right]=\frac{1}{n_{i}}V\left[G_{i}^{\prime }\right]=\frac{1}{n_{i}}{\sigma^{\prime }}_{i}^{2}$.

**Theorem** **1.** *For any set of weights ${\left\{\alpha_{i}\right\}}_{i=1}^{n}$, such as $\sum_{i=1}^{n}\alpha_{i}=1$ and any set of weights ${\left\{\beta_{i}\right\}}_{i=1}^{n}$, such as $\sum_{i=1}^{n}\beta_{i}=1$, G is an unbiased estimator of μ.*

**Proof.**  The estimator *G* is unbiased, since

(21)E[G]=∑i=1nαiE[Gi]=∑i=1nαiμi′=∑iαi∫f(x)pi(x)∑k=1nαkpk(x)dx(22)=∫f(x)∑i=1nαipi(x)∑k=1nαkpk(x)dx=∫f(x)dx≡μ.

□

The variance of *G* is given by

(23) $$\begin{array}{c} V\left[G\right]=V\left[\sum_{i=1}^{n}\alpha_{i}G_{i}\right]=\sum_{i=1}^{n}\alpha_{i}^{2}V\left[G_{i}\right]=\sum_{i=1}^{n}\frac{\alpha_{i}^{2}{\sigma^{\prime }}_{i}^{2}}{n_{i}}. \end{array}$$

For the sake of the theoretical analysis, we define $G^{1}$, a normalized version of *G* with N=1 (see Section 2.1), with variance

(24) $$\begin{array}{c} V\left[G^{1}\right]=\sum_{i=1}^{n}\frac{\alpha_{i}^{2}{\sigma^{\prime }}_{i}^{2}}{\beta_{i}}. \end{array}$$

Next we study four special cases of estimator $G^{1}$.

**Remark** **1.** *We could also consider the one-sample estimator G, randomized version of G. However, G is a particular case of the general estimator Z, and we have seen in Section 2.2 that the optimal case for Z is when Z≡F, thus it only makes sense to consider the extension G of the multi-sample estimator F.*

**Remark** **2.** *Grittmann et al. estimator [19], can be interpreted as a G estimator where $\alpha_{i}\propto \beta_{i}\frac{m_{i}^{2}}{v_{i}}=\beta_{i}\frac{v_{i}+\mu^{2}}{v_{i}}$, where $v_{i}$ is the variance of i independent technique and $m_{i}^{2}=v_{i}+\mu^{2}$ the second moment,*

(25) $$v_{i}=\int \frac{f{\left(x\right)}^{2}}{p_{i}\left(x\right)}dx.$$

### 3.1. Case 1: $\alpha_{i}=\beta_{i}$, ∀i

In this particular case, the estimator *G* reverts to *F*. The variance is

(26) $$\begin{array}{c} V\left[F^{1}\right]=\sum_{i=1}^{n}\alpha_{i}{\sigma^{\prime }}_{i}^{2}, \end{array}$$

by simple substitution in Equation (24). We aim at finding the optimal ${\left\{\alpha_{i}^{*}\right\}}_{i=1}^{n}$ such the variance of Equation (26) is minimized.

**Theorem** **2.** The optimal estimator $F^{*}$ in terms of variance is achieved when ∀j∈{1,…,n}, the following values are equal

(27) $$\begin{array}{c} {\sigma^{\prime }}_{j}^{2}+2{\mu_{j}^{\prime }}^{2}-2\sum_{i=1}^{n}\alpha_{i}^{*}\mu_{i}^{\prime }\int \frac{f\left(x\right)p_{i}\left(x\right)p_{j}\left(x\right)}{{\left(\sum_{k=1}^{n}\alpha_{k}^{*}p_{k}\left(x\right)\right)}^{2}}dx. \end{array}$$

See Appendix B for a proof.

Compare Equation (27) with the condition for optimal $F^{*}$, which is [9] that the following expression is equal ∀j∈{1,…,n},

(28) $$\begin{array}{c} {\sigma^{\prime }}_{j}^{2}+{\mu_{j}^{\prime }}^{2}. \end{array}$$

The optimal ${\left\{\alpha_{i}^{*}\right\}}_{i=1}^{n}$ solutions are in general different for *F*, Equation (27), and for $F^{*}$, Equation (28), although observe that, when for ${\left\{\alpha_{i}^{*}\right\}}_{i=1}^{n}$ all *i* the $\mu_{i}^{\prime }$ are equal, then the optimality condition Equation (27) implies that ∀i∈{1,…,n} the ${\sigma^{\prime }}_{i}^{2}$ are equal too, and thus the optimal solutions for *F* and F are the same, in concordance with being V[F]=V[F] for this special case, see Section 2.2 and Appendix A.

Another interesting result regarding the $\mu_{i}^{\prime }$ values is the following theorem,

**Theorem** **3.** *If V[F(F)]=0 then for all i, $\mu_{i}^{\prime }=\mu$.*

**Proof.**  As V[F]≤V[F(F)] then $V\left[F\right]=\sum_{i}^{n}\alpha_{i}{\sigma_{i}^{\prime }}^{2}=0$, and thus for all *i*, $\sigma_{i}^{\prime }=0$; however, V[F(F)]=0 is obviously a local (and global) minimum for V[F(F)] (a convex function) and thus from Equation (28) for all *i*, ${\sigma^{\prime }}_{i}^{2}+{\mu_{i}^{\prime }}^{2}$ are equal and hence the result. Alternatively, we know that V[F]=V[F(F)]⇔ for all *i*, all $\mu_{i}^{\prime }=\mu$. □

### 3.2. Case 2: Fixed ${\left\{\alpha_{i}\right\}}_{i=1}^{n}$

Consider now that ${\left\{\alpha_{i}\right\}}_{i=1}^{n}$ are fixed, and hence also ${\left\{{\sigma^{\prime }}_{i}^{2}\right\}}_{i=1}^{n}$, are fixed. Applying Cauchy–Schwartz inequality to sequences ${\left\{\sqrt{\beta_{i}}\right\}}_{i=1}^{n}$ and ${\left\{\frac{\alpha_{i}{\sigma^{\prime }}_{i}}{\sqrt{\beta_{i}}}\right\}}_{i=1}^{n}$ we obtain

(29) $$\begin{array}{c} {\left(\sum_{i=1}^{n}\alpha_{i}\sigma_{i}^{\prime }\right)}^{2}\leq \left(\sum_{i=1}^{n}\beta_{i}\right)\left(\sum_{i=1}^{n}\frac{\alpha_{i}^{2}{\sigma^{\prime }}_{i}^{2}}{\beta_{i}}\right). \end{array}$$

As the left term is fixed, equality in Equation (29) will give the minimum of right term, being this term $V[G^{1}]$ as $\sum_{i=1}^{n}\beta_{i}=1$; however, equality can only happen when the right term sequences are proportional, this is, for all *i*, $\sqrt{\beta_{i}}\propto \frac{\alpha_{i}{\sigma^{\prime }}_{i}}{\sqrt{\beta_{i}}}$, and thus the optimal ${\left\{\beta_{i}\right\}}_{i=1}^{n}$ are given by

(30) $$\begin{array}{c} \beta_{i}^{*}\propto \alpha_{i}\sigma_{i}^{\prime },\;i=1,...n, \end{array}$$

and the optimal (minimum) variance is

(31) $$\begin{array}{c} V\left[G^{1*}\right]={\left(\sum_{i=1}^{n}\alpha_{i}\sigma_{i}^{\prime }\right)}^{2}. \end{array}$$

**Theorem** **4.** Given an estimator F with ${\left\{\alpha_{i}\right\}}_{i=1}^{n}$ values, we can always find a better estimator G by sampling as $\beta_{i}^{*}\propto \alpha_{i}\sigma_{i}^{\prime }$, which is strictly better whenever not all ${\sigma^{\prime }}_{i}$ are equal.

**Proof.**  Observe that, by applying Cauchy–Schwartz inequality to the sequences ${\left\{\sqrt{\alpha_{i}}\right\}}_{i=1}^{n}$ and ${\left\{\sqrt{\alpha_{i}}{\sigma^{\prime }}_{i}\right\}}_{i=1}^{n}$,

(32) $$\begin{array}{c} {\left(\sum_{i=1}^{n}\alpha_{i}\sigma_{i}^{\prime }\right)}^{2}\leq \left(\sum_{i=1}^{n}\alpha_{i}\right)\left(\sum_{i=1}^{n}\alpha_{i}{\sigma^{\prime }}_{i}^{2}\right), \end{array}$$

but the left-hand side of inequality is $V[G^{1*}]$, and as $\sum_{i=1}^{n}\alpha_{i}=1$, the right-hand side is $V[F^{1}]$. Hence, for the optimal values ${\left\{\beta_{i}^{*}\right\}}_{i=1}^{n}$ as in Equation (30), the estimator $G^{*}$ always outperforms the estimator *F* (When comparing estimators *F* and *G*, we consider, unless explicitly stated, the same set of ${\left\{\alpha_{i}\right\}}_{i=1}^{n}$ values).

Equality in Equation (32) only happens when for all *i*, $\sqrt{\alpha_{i}}\propto \sqrt{\alpha_{i}}{\sigma^{\prime }}_{i}$, i.e., when all ${\sigma^{\prime }}_{i}$ are equal. In that case we have for all *i*$\beta_{i}^{*}=\alpha_{i}$, and we revert to the *F* estimator. □

**Remark** **3.** From the inequality $\left(\sum_{i=1}^{n}\frac{1}{n}{\sigma^{\prime }}_{i}^{2}\right)\leq n{\left(\sum_{i=1}^{n}\frac{1}{n}\sigma_{i}^{\prime }\right)}^{2}$, the maximum possible acceleration by using the optimal $\beta_{i}^{*}$ values when for all i, $\alpha_{i}=1/n$, is equal to n (as observed in [7]). This acceleration would be approached when there is an index k such that for all i≠k, $\sigma_{k}^{\prime }>>\sigma_{i}^{\prime }$. In general, the more different the $\sigma_{i}^{\prime }$ are, the higher the acceleration.

A particular case of Theorem 4 is when $\alpha_{i}=\frac{1}{n}$, ∀i, where $V[G^{1}]$ becomes

(33) $$\begin{array}{c} V\left[G^{1}\right]=\frac{1}{n^{2}}\sum_{i=1}^{n}\frac{{\sigma^{\prime }}_{i}^{2}}{\beta_{i}}. \end{array}$$

This case was introduced in Section 4 of [7]. It was shown that this estimator is provably better than *F* with $\alpha_{i}=1/n,\forall i$ when

(34) $$\begin{array}{c} \beta_{i}^{*}\propto \sigma_{i}^{\prime },\;i=1,\ldots ,n, \end{array}$$

which is the optimal case of Equation (33). Examples showing the improvement obtained were also given in [7].

#### Optimal Efficiency

Let us now take into account that the cost of each sampling technique can be different, as it is usually considered in the literature [25]. Let us denote the cost of sampling technique *i* as $c_{i}$. The inverse of efficiency for the estimator *G* is given by

(35) $$\begin{array}{c} E_{G}^{-1}=\left(\sum_{i=1}^{n}\beta_{i}c_{i}\right)\left(\sum_{i=1}^{n}\frac{\alpha_{i}^{2}{\sigma^{\prime }}_{i}^{2}}{\beta_{i}}\right). \end{array}$$

Note that this quantity represents the total cost multiplied by the variance of the estimator. Using Cauchy–Schwartz inequality with the sequences ${\left\{\sqrt{\beta_{i}c_{i}}\right\}}_{i=1}^{n}$ and ${\left\{\frac{\alpha_{i}{\sigma^{\prime }}_{i}}{\sqrt{\beta_{i}}}\right\}}_{i=1}^{n}$, we obtain

(36) $$\begin{array}{c} {\left(\sum_{i=1}^{n}\alpha_{i}\sigma_{i}^{\prime }\sqrt{c_{i}}\right)}^{2}\leq \left(\sum_{i=1}^{n}\beta_{i}c_{i}\right)\left(\sum_{i=1}^{n}\frac{\alpha_{i}^{2}{\sigma^{\prime }}_{i}^{2}}{\beta_{i}}\right). \end{array}$$

The optimal sampling rates (for maximizing the efficiency) are those that yield Equation (36) as an equality, which happens when $\beta_{i}^{*}\propto \frac{\alpha_{i}{\sigma^{\prime }}_{i}}{\sqrt{c_{i}}}$. Observe that, using again the Cauchy–Schwartz theorem, with sequences ${\left\{\sqrt{\alpha_{i}c_{i}}\right\}}_{i=1}^{n}$ and ${\left\{\sqrt{\alpha_{i}}{\sigma^{\prime }}_{i}\right\}}_{i=1}^{n}$, we obtain

(37) $$\begin{array}{c} {\left(\sum_{i=1}^{n}\alpha_{i}\sigma_{i}^{\prime }\sqrt{c_{i}}\right)}^{2}\leq \left(\sum_{i=1}^{n}\alpha_{i}c_{i}\right)\left(\sum_{i=1}^{n}\alpha_{i}{\sigma^{\prime }}_{i}^{2}\right), \end{array}$$

where the left-hand side is $E_{G}^{-1}$ with the optimal sampling rates, and the right-hand side is $E_{F}^{-1}$. Note that equality only happens when for all *i*, $\sqrt{\alpha_{i}c_{i}}=\sqrt{\alpha_{i}}{\sigma^{\prime }}_{i}$, i.e., $c_{i}\propto {\sigma^{\prime }}_{i}^{2}$, where we have $\beta_{i}^{*}=\alpha_{i}$ and we revert to the *F* estimator. This is summarized in the following theorem.

**Theorem** **5.** Given an estimator F with ${\left\{\alpha_{i}\right\}}_{i=1}^{n}$ values, and sampling costs ${\left\{c_{i}\right\}}_{i=1}^{n}$, we can always find a strictly more efficient estimator $G^{*}$ when for all i, $\beta_{i}^{*}\propto \alpha_{i}\frac{\sigma_{i}^{\prime }}{\sqrt{c_{i}}}$, and not all $c_{i}\propto {\sigma^{\prime }}_{i}^{2}$.

### 3.3. Case 3: Fixed ${\left\{\beta_{i}\right\}}_{i=1}^{n}$

**Theorem** **6.** Consider now a fixed set ${\left\{\beta_{i}\right\}}_{i=1}^{n}$. The optimal set ${\left\{\alpha_{i}^{*}\right\}}_{i=1}^{n}$ can be found using Lagrange multipliers with target function

$$\Lambda \left({\left\{\alpha_{i}\right\}}_{i=1}^{n},\lambda \right)=\sum_{i=1}^{n}\frac{\alpha_{i}^{2}{\sigma^{\prime }}_{i}^{2}}{\beta_{i}}+\lambda \left(\sum_{i=1}^{n}\alpha_{i}-1\right).$$

*Observe that the ${\sigma^{\prime }}_{i}^{2}$ values depend on the ${\left\{\alpha_{i}\right\}}_{i=1}^{n}$ values. The optimal values are those that obey, for all j, the following expression*

(38) $$\begin{array}{cc} \; & \frac{\alpha_{j}^{*}{\sigma^{\prime }}_{j}^{2}}{\beta_{j}}=\sum_{i=1}^{n}\frac{\alpha_{i}^{*2}}{\beta_{i}}\times \\ \; & \left(\int \frac{f^{2}\left(x\right)p_{i}\left(x\right)p_{j}\left(x\right)}{{\left(\sum_{k=1}^{n}\alpha_{k}^{*}p_{k}\left(x\right)\right)}^{3}}dx-\mu_{i}^{\prime }\int \frac{f\left(x\right)p_{i}\left(x\right)p_{j}\left(x\right)}{{\left(\sum_{k=1}^{n}\alpha_{k}^{*}p_{k}\left(x\right)\right)}^{2}}dx\right). \end{array}$$

**Proof.**  The derivation can be found in the Appendix C. □

Note that in the general case, the optimal $\alpha_{i}^{*}\neq \beta_{i}$.

Moreover, aside from the optimal values ${\left\{\alpha_{j}^{*}\right\}}_{j=1}^{n}$, we can find cases where $V\left[G^{1}\right]\leq V\left[F^{1}\right]$. Given ${\left\{\beta_{i}\right\}}_{i=1}^{n}$, from Theorem 4 we have that if there exist ${\left\{\alpha_{i}\right\}}_{i=1}^{n}$ such that $\beta_{i}\propto \alpha_{i}\sigma_{i}^{\prime },\;i=1,...n$, then $V\left[G^{1}\right]\leq V\left[F^{1}\right]$. From Theorem 5, we have that if there exist ${\left\{\alpha_{i}\right\}}_{i=1}^{n}$ such that $\beta_{i}\propto \alpha_{i}\frac{\sigma_{i}^{\prime }}{\sqrt{c_{i}}},\;\forall i=1,...n$, then the estimator $G^{1}$ is more efficient than estimator $F^{1}$.

Observe that from Equation (38), a necessary and sufficient condition for the optimal solution $\alpha_{i}^{*}$ to be such that $\alpha_{i}^{*}=\beta_{i}$ for all *i* is that Equation (39) holds (see Appendix C),

(39) $$\begin{array}{c} {\mu_{j}^{\prime }}^{2}=\sum_{i=1}^{n}\alpha_{i}\mu_{i}^{\prime }\int \frac{f\left(x\right)p_{i}\left(x\right)p_{j}\left(x\right)}{{\left(\sum_{k=1}^{n}\alpha_{k}p_{k}\left(x\right)\right)}^{2}}dx, \end{array}$$

which is satisfied when for all *i*, $\mu_{i}^{\prime }=\mu$. In this particular case, $\mu_{i}^{\prime }=\mu$, G≡F, and V[G]=V[F]=V[F]. In other words, when choosing a sampling rate of $\left\{\beta_{i}\right\}=\left\{\alpha_{i}^{⋆}\right\}$, where $\left\{\alpha_{i}^{⋆}\right\}$ are such that for all *i*, $\mu_{i}^{\prime }=\mu$, the optimal $\left\{\alpha_{i}\right\}$ parameters for *G* estimator are precisely $\left\{\alpha_{i}^{⋆}\right\}$. That is, we can not improve the $F(\left\{\alpha_{i}^{⋆}\right\})$ estimator with the *G* estimator with a suitable selection of $\left\{\beta_{i}\right\}$ parameters. Observe that it is similar to the case studied in Section 3.2 for the $\left\{\alpha_{i}\right\}$ that make all ${\sigma^{\prime }}_{i}$ equal.

Equation (39) might have additional solutions to the one (if exists) that makes all $\mu_{i}^{\prime }$ equal, in this case V[G]=V[F] too but V[F]≠V[F].

### 3.4. Case 4: $\beta_{i}=1/n,\forall i$

In the case when for all *i*, $\beta_{i}=1/n$, the variance becomes

(40) $$\begin{array}{c} V\left[G^{1}\right]=\sum_{i=1}^{n}\frac{\alpha_{i}^{2}{\sigma^{\prime }}_{i}^{2}}{1/n}=n\sum_{i=1}^{n}\alpha_{i}^{2}{\sigma^{\prime }}_{i}^{2}. \end{array}$$

Note that this is a usual case in the MIS literature strategies [2,4,5,6] and the adaptive IS (AIS) literature [11,12,13,14,15,24], since all the techniques have the same number of counts. By setting in Equation (38) for all *i*, $\beta_{i}=1/n$, and if we can optimize ${\left\{\alpha_{j}\right\}}_{j=1}^{n}$, we can find the minimum variance values ${\left\{\alpha_{j}^{*}\right\}}_{j=1}^{n}$. Thus the minimum variance $V[G^{*}]$ corresponds, if the solution exists, to the values ${\left\{\alpha_{j}^{*}\right\}}_{j=1}^{n}$ that satisfy

(41) $$\begin{array}{cc} \; & \alpha_{j}^{*}{\sigma^{\prime }}_{j}^{2}=\sum_{i=1}^{n}\alpha_{i}^{*2}\\ \; & \times \left(\int \frac{f^{2}\left(x\right)p_{i}\left(x\right)p_{j}\left(x\right)}{{\left(\sum_{k=1}^{n}\alpha_{k}^{*}p_{k}\left(x\right)\right)}^{3}}dx-\mu_{i}^{\prime }\int \frac{f\left(x\right)p_{i}\left(x\right)p_{j}\left(x\right)}{{\left(\sum_{k=1}^{n}\alpha_{k}^{*}p_{k}\left(x\right)\right)}^{2}}dx\right). \end{array}$$

The corresponding variance $V[G^{*}]$ will be less or equal than the variance of V[G] for all ${\left\{\alpha_{j}\right\}}_{j=1}^{n}$, and in particular for $\alpha_{i}=1/n$, where *G* converts into *F* as $\beta_{i}=\alpha_{i}=1/n$, the classic balance heuristic estimator, thus $V\left[G^{*}\left(\left\{\alpha_{i}^{*}\right\},\left\{\beta_{i}=1/n\right\}\right)\right]\leq V\left[F\left(\left\{\alpha_{i}=1/n\right\}\right)\right]$. Observe that in general $\left\{\alpha_{i}^{*}\neq 1/n\right\}$, because substituting $\left\{\alpha_{i}^{*}=1/n\right\}$ in Equation (41) the resulting equation Equation (39) is not satisfied in general.

#### Comparing $V[G^{1}\left(\left\{\alpha_{i}\right\},\left\{\beta_{i}\right\}\right)]$, $V[G^{1}\left(\left\{\alpha_{i}\right\},\left\{\beta_{i}=1/n\right\}\right)]$ and $V[F^{1}\left(\left\{\alpha_{i}\right\}\right)]$

Let us see first when $V\left[G^{1}\left(\left\{\alpha_{i}\right\},\left\{\beta_{i}\right\}\right)\right]\leq V\left[G^{1}\left(\left\{\alpha_{i}\right\},\left\{\beta_{i}=1/n\right\}\right)\right)]$ and viceversa.

**Theorem** **7.** *If the sequences $\left\{\beta_{i}\right\},\left\{\frac{\alpha_{i}^{2}{\sigma^{\prime }}_{i}^{2}}{\beta_{i}}\right\}$ are comonotonic, i.e., $\beta_{i}\leq \beta_{j}\Rightarrow \frac{\alpha_{i}^{2}{\sigma^{\prime }}_{i}^{2}}{\beta_{i}}\leq \frac{\alpha_{j}^{2}{\sigma^{\prime }}_{j}^{2}}{\beta_{j}}$, then*

(42) $$\begin{array}{c} V\left[G^{1}\left(\left\{\alpha_{i}\right\},\left\{\beta_{i}\right\}\right)\right]\leq V\left[G^{1}\left(\left\{\alpha_{i}\right\},\left\{\beta_{i}=1/n\right\}\right)\right]. \end{array}$$

**Proof.**  Applying Theorem 9, Corollary 5 from [26],

(43) $$\begin{array}{ccc} \sum_{i=1}^{n}\frac{1}{n}\left(\frac{\alpha_{i}^{2}{\sigma^{\prime }}_{i}^{2}}{\beta_{i}}\right) & \leq & \sum_{i=1}^{n}\beta_{i}\left(\frac{\alpha_{i}^{2}{\sigma^{\prime }}_{i}^{2}}{\beta_{i}}\right), \end{array}$$

obtaining the inequality in Equation (42). See also [10,27]. □

Observe that equality in Equation (43) happens when for all *i*, $\beta_{i}\propto \alpha_{i}^{2}{\sigma^{\prime }}_{i}^{2}$.

In the same way we can proof the inverse case as follows,

**Theorem** **8.** *If the sequences $\left\{\beta_{i}\right\}$ and $\left\{\frac{\alpha_{i}^{2}{\sigma^{\prime }}_{i}^{2}}{\beta_{i}}\right\}$ are countermonotonic, i.e., $\beta_{i}\leq \beta_{j}\Rightarrow \frac{\alpha_{i}^{2}{\sigma^{\prime }}_{i}^{2}}{\beta_{i}}\geq \frac{\alpha_{j}^{2}{\sigma^{\prime }}_{j}^{2}}{\beta_{j}}$, then*

(44) $$\begin{array}{c} V\left[G^{1}\left(\left\{\alpha_{i}\right\},\left\{\beta_{i}\right\}\right)\right]\geq V\left[G^{1}\left(\left\{\alpha_{i}\right\},\left\{\beta_{i}=1/n\right\}\right)\right)]. \end{array}$$

The proof implies for countermonotonicity a change of sign in the inequality in Theorem 9, Corollary 5 from [26].

Let us compare now $V[G^{1}\left(\left\{\alpha_{i}\right\},\left\{\beta_{i}=1/n\right\}\right)]$ and $V[F^{1}\left(\left\{\alpha_{i}\right\}\right)]$.

**Theorem** **9.** *If the sequences $\left\{\alpha_{i}\right\},\left\{\alpha_{i}{\sigma^{\prime }}_{i}^{2}\right\}$ are countermonotonic, i.e., $\alpha_{i}\leq \alpha_{j}\Rightarrow \alpha_{i}{\sigma^{\prime }}_{i}^{2}\geq \alpha_{j}{\sigma^{\prime }}_{j}^{2}$, then*

(45) $$\begin{array}{c} V\left[G^{1}\left(\left\{\alpha_{i}\right\},\left\{\beta_{i}=1/n\right\}\right)\right]\leq V\left[F^{1}\left(\left\{\alpha_{i}\right\}\right)\right]. \end{array}$$

**Proof.**  The proof is as above, making use of Theorem 9, Corollary 5 from [26],

(46) $$\begin{array}{ccc} \sum_{i=1}^{n}\frac{1}{n}\left(\alpha_{i}{\sigma^{\prime }}_{i}^{2}\right) & \geq & \sum_{i=1}^{n}\alpha_{i}\left(\alpha_{i}{\sigma^{\prime }}_{i}^{2}\right), \end{array}$$

obtaining the inequality in Equation (45). □

Observe that equality in Equation (46) happens when for all *i*, $\alpha_{i}\propto 1/{\sigma^{\prime }}_{i}^{2}$. In the same way we can prove the reverse case of Theorem 9.

**Theorem** **10.** *If the sequences $\left\{\alpha_{i}\right\},\left\{\alpha_{i}{\sigma^{\prime }}_{i}^{2}\right\}$ are comonotonic, i.e., $\alpha_{i}\leq \alpha_{j}\Rightarrow \alpha_{i}{\sigma^{\prime }}_{i}^{2}\leq \alpha_{j}{\sigma^{\prime }}_{j}^{2}$, then*

(47) $$\begin{array}{c} V\left[G^{1}\left(\left\{\alpha_{i}\right\},\left\{\beta_{i}=1/n\right\}\right)\right]\geq V\left[F^{1}\left(\left\{\alpha_{i}\right\}\right)\right]. \end{array}$$

As a direct consequence of Theorems 9 and 10 we have the following corollary:

**Corollary** **1.** *When $\alpha_{i}\propto 1/{\sigma^{\prime }}_{i}^{2+\epsilon }$, if −2≤ϵ≤0 then $V\left[G^{1}\left(\left\{\alpha_{i}\right\},\left\{\beta_{i}=1/n\right\}\right)\right]\leq V\left[F^{1}\left(\left\{\alpha_{i}\right\}\right)\right]$ holds, and if ϵ≥0 then $V\left[G^{1}\left(\left\{\alpha_{i}\right\},\left\{\beta_{i}=1/n\right\}\right)\right]\geq V\left[F^{1}\left(\left\{\alpha_{i}\right\}\right)\right]$ holds.*

By setting ϵ=−1 in Corollary 1, the products $\alpha_{i}{\sigma^{\prime }}_{i}$ are equal for all *i*, which is equivalent to saying that $\alpha_{i}{\sigma^{\prime }}_{i}\propto 1/n=\beta_{i}$. Observe that this corresponds to the optimal solution $\left\{\beta_{j}^{*}\right\}$ given by Equation (30). Observe now that the inequality for this optimal solution, $V\left[G^{1}\right]\leq V\left[F^{1}\right]$, can be obtained by successive application of Theorems 7 and 9.

**Theorem** **11.** *If the sequences $\left\{\alpha_{i}\right\},\left\{\alpha_{i}{\sigma^{\prime }}_{i}^{2}\right\}$ are countermonotonic, and the sequences $\left\{\beta_{i}\right\},\left\{\frac{\alpha_{i}^{2}{\sigma^{\prime }}_{i}^{2}}{\beta_{i}}\right\}$ are comonotonic, then*

(48) $$\begin{array}{c} V\left[G^{1}\left(\left\{\alpha_{i}\right\},\left\{\beta_{i}\right\}\right)\right]\leq V\left[F^{1}\left(\left\{\alpha_{i}\right\}\right)\right]. \end{array}$$

**Proof.**  The result is obtained by successive application of Theorems 7 and 9. □

**Theorem** **12.** *If the sequences $\left\{\alpha_{i}\right\},\left\{\alpha_{i}{\sigma^{\prime }}_{i}^{2}\right\}$ are comonotonic, and the sequences $\left\{\beta_{i}\right\},\left\{\frac{\alpha_{i}^{2}{\sigma^{\prime }}_{i}^{2}}{\beta_{i}}\right\}$ are countermonotonic, then*

(49) $$\begin{array}{c} V\left[G^{1}\left(\left\{\alpha_{i}\right\},\left\{\beta_{i}\right\}\right)\right]\geq V\left[F^{1}\left(\left\{\alpha_{i}\right\}\right)\right]. \end{array}$$

As a direct consequence of Theorems 11 and 12 we have the following corollary:

**Corollary** **2.** *When $\alpha_{i}\propto 1/{\sigma^{\prime }}_{i}^{2+\epsilon }$ and $\beta_{i}\propto {\left(\alpha_{i}{\sigma^{\prime }}_{i}\right)}^{\gamma }$, if −2≤ϵ≤0 and 0<γ≤2 then $V\left[G^{1}\left(\left\{\alpha_{i}\right\},\left\{\beta_{i}\right\}\right)\right]\leq V\left[F^{1}\left(\left\{\alpha_{i}\right\}\right)\right]$ holds, and if ϵ≥0 and γ>2 then $V\left[G^{1}\left(\left\{\alpha_{i}\right\},\left\{\beta_{i}\right\}\right)\right]\geq V\left[F^{1}\left(\left\{\alpha_{i}\right\}\right)\right]$ holds.*

For example, take $\alpha_{i}\propto 1/{\sigma^{\prime }}_{i}^{2}$, and $\beta_{i}\propto \alpha_{i}{\sigma^{\prime }}_{i}\propto 1/{\sigma^{\prime }}_{i}$, then $V\left[G^{1}\left(\left\{\alpha_{i}\right\},\left\{\beta_{i}\right\}\right)\right]\leq V\left[F^{1}\left(\left\{\alpha_{i}\right\}\right)\right].$

Finally, observe that we do not have a sufficient condition for $V\left[F^{1}\left(\left\{\alpha_{i}\right\}\right)\right]\leq V\left[F^{1}\left(\left\{\alpha_{i}=1/n\right\}\right)\right]$, only there are heuristics [7,9,10,28].

### 3.5. Case 5: General Case

Let us consider the global optimum for *G* estimator, this is, without imposing any constraint to the parameters $\left\{\alpha_{i}\right\},\left\{\beta_{i}\right\}$. For this we should optimize the lagrangian function

$$\Lambda \left({\left\{\alpha_{i}\right\}}_{i=1}^{n},{\left\{\beta_{i}\right\}}_{i=1}^{n},\lambda_{1},\lambda_{2}\right)=\sum_{i=1}^{n}\frac{\alpha_{i}^{2}{\sigma^{\prime }}_{i}^{2}}{\beta_{i}}+\lambda_{1}\left(\sum_{i=1}^{n}\alpha_{i}-1\right)+\lambda_{2}\left(\sum_{i=1}^{n}\beta_{i}-1\right).$$

Differentiating with respect to $\alpha_{i}$,

$$\frac{\partial \Lambda ({\left\{\alpha_{i}\right\}}_{i=1}^{n},{\left\{\beta_{i}\right\}}_{i=1}^{n},\lambda_{1},\lambda_{2})}{\partial_{\alpha_{j}}}=0,$$

we obtain Equation (38).

Differentiating with respect to $\beta_{i}$,

$$\frac{\partial \Lambda ({\left\{\alpha_{i}\right\}}_{i=1}^{n},{\left\{\beta_{i}\right\}}_{i=1}^{n},\lambda_{1},\lambda_{2})}{\partial_{\beta_{j}}}=0,$$

we obtain Equation (30). Thus, the optimal parameters ${\left\{\alpha_{i}^{⋆}\right\}}_{i=1}^{n}$ and ${\left\{\beta_{i}^{⋆}\right\}}_{i=1}^{n}$ will solve for Equations (38) and (30) simultaneously.

Let us consider now the ${\left\{\alpha_{i}^{†}\right\}}_{i=1}^{n}$, if they exist, such that for all *i*, $\sigma_{i}^{\prime }$ are equal. Then, for Equation (30) to hold for ${\left\{\alpha_{i}^{†}\right\}}_{i=1}^{n}$, we have that $\beta_{i}^{†}=\alpha_{i}^{†}$ for all i=1,…,n. From Equation (38), taking ${\left\{\beta_{i}^{†}\right\}}_{i=1}^{n}={\left\{\alpha_{i}^{†}\right\}}_{i=1}^{n}$, Equation (39) has to hold for all $\alpha_{i}^{†}$.

Furthermore, the holding of Equation (39), together with the hypothesis that for all *j*, ${\sigma^{\prime }}_{j}^{2}$ are equal, allows Equation (27) to hold, and then ${\left\{\alpha_{i}^{†}\right\}}_{i=1}^{n}$ is an optimum for *F* too, V[F]=V[G]. If in addition, if $\left\{\alpha_{i}^{†}\right\}$ are such that they make all ${\left\{\mu^{\prime }\right\}}_{j=1}^{n}$ equal, then they would be optimal for F too, V[F]=V[F]=V[G].

## 4. Singular Solutions

The variance of F can be written as [7]

(50) $$\begin{array}{ccc} V[F] & = & \sum_{i=1}^{n}\alpha_{i}\left({\sigma^{\prime }}_{i}^{2}+{\mu^{\prime }}_{i}^{2}-\mu^{2}\right)=\sum_{i=1}^{n}\alpha_{i}{\sigma^{\prime }}_{i}^{2}+\sum_{i=1}^{n}\alpha_{i}{\mu^{\prime }}_{i}^{2}-\mu^{2}.\\ & = & \sum_{i=1}^{n}\alpha_{i}\left({\sigma^{\prime }}_{i}^{2}+{\mu^{\prime }}_{i}^{2}\right)-\mu^{2}. \end{array}$$

The optimal parameters ${\left\{\alpha_{i}\right\}}_{i=1}^{n}$ happen when for all *i*, ${\sigma^{\prime }}_{i}^{2}+{\mu^{\prime }}_{i}^{2}$ equal, which gives a local minimum for V[F] (in this case a global minimum too, as V[F] is convex). Observe that this makes, in the third inequality, all terms factored by $\left\{\alpha_{i}\right\}$ equal, and also corresponds to equality in the Cauchy–Schwartz inequality

(51) $$\begin{array}{c} {\left(\sum_{i=1}^{n}\alpha_{i}\sqrt{\left({\sigma^{\prime }}_{i}^{2}+{\mu^{\prime }}_{i}^{2}\right)}\right)}^{2}\leq \left(\sum_{i=1}^{n}\alpha_{i}\right)\left(\sum_{i=1}^{n}\alpha_{i}\left({\sigma^{\prime }}_{i}^{2}+{\mu^{\prime }}_{i}^{2}\right)\right), \end{array}$$

with equality only when, for all *i*, ${\sigma^{\prime }}_{i}^{2}+{\mu^{\prime }}_{i}^{2}$ are equal.

If there exist ${\left\{\alpha_{i}^{⋆}\right\}}_{i=1}^{n}$ values such that for all *i*, $\mu_{i}^{\prime }=\mu$ and all ${\sigma^{\prime }}_{i}$ are equal, these values correspond to the global optimum $V\left[F^{⋆}\right]=V\left[F^{⋆}\right]=V\left[G^{⋆}\right]$, and $F^{⋆}\equiv G^{⋆}$, but $F^{⋆}≢F^{⋆}$. Suppose there exist ${\left\{\alpha_{i}^{⋆}\right\}}_{i=1}^{n}$ such that for all *i*, $\mu_{i}^{\prime }=\mu$, then we can not improve the *F* estimator with a *G* estimator with a suitable selection of ${\left\{\beta_{i}\right\}}_{i=1}^{n}$ parameters, because the optimal $\left\{\alpha_{i}\right\}$ parameters for the *G* estimator when ${\left\{\beta_{i}\right\}}_{i=1}^{n}={\left\{\alpha_{i}^{⋆}\right\}}_{i=1}^{n}$ are precisely $\left\{\alpha_{i}^{⋆}\right\}$. This is, the optimal of $V[G\left({\left\{\alpha_{i}\right\}}_{i=1}^{n},{\left\{\beta_{i}\right\}}_{i=1}^{n}={\left\{\alpha_{i}^{⋆}\right\}}_{i=1}^{n}\right)]$ is for ${\left\{\alpha_{i}\right\}}_{i=1}^{n}={\left\{\alpha_{i}^{⋆}\right\}}_{i=1}^{n}$.

Let us see now V[F]:

$$V\left[F\right]=\sum_{i=1}^{n}\alpha_{i}{\sigma^{\prime }}_{i}^{2}.$$

Suppose there exist ${\left\{\alpha_{i}^{⋆}\right\}}_{i=1}^{n}$ such that all $\sigma_{i}^{\prime }$ are equal, then we can not improve the *F* estimator with a suitable selection of ${\left\{\beta_{i}\right\}}_{i=1}^{n}$ parameters for the *G* estimator, because the optimal ${\left\{\beta_{i}^{⋆}\right\}}_{i=1}^{n}$ are such that ${\left\{\beta_{i}^{⋆}\right\}}_{i=1}^{n}={\left\{\alpha_{i}^{⋆}\right\}}_{i=1}^{n}$. This is, the optimal of $V[G\left({\left\{\alpha_{i}^{⋆}\right\}}_{i=1}^{n},{\left\{\beta_{i}\right\}}_{i=1}^{n}\right)]$ is for ${\left\{\beta_{i}\right\}}_{i=1}^{n}={\left\{\alpha_{i}^{⋆}\right\}}_{i=1}^{n}$. Observe that by Cauchy–Schwartz

(52) $$\begin{array}{c} {\left(\sum_{i=1}^{n}\alpha_{i}{\sigma^{\prime }}_{i}\right)}^{2}\leq \left(\sum_{i=1}^{n}\alpha_{i}\right)\left(\sum_{i=1}^{n}\alpha_{i}{\sigma^{\prime }}_{i}^{2}\right), \end{array}$$

with equality only when, for all *i*, all the ${\sigma^{\prime }}_{i}^{2}$ are equal. Observe thus that both the solutions for $\mu_{i}^{\prime }$ equal and for ${\sigma^{\prime }}_{i}^{2}$ equal are singular solutions for *G*, where it reverts to *F*.

Let us consider now V[G]:

(53) $$\begin{array}{c} V\left[G\right]=\sum_{i=1}^{n}\frac{\alpha_{i}^{2}}{\beta_{i}}{\sigma^{\prime }}_{i}^{2}=\sum_{i=1}^{n}\alpha_{i}\left(\frac{\alpha_{i}}{\beta_{i}}{\sigma^{\prime }}_{i}^{2}\right)=\sum_{i=1}^{n}\beta_{i}\left(\frac{\alpha_{i}^{2}}{\beta_{i}^{2}}{\sigma^{\prime }}_{i}^{2}\right). \end{array}$$

In the second equality in Equation (53), the solution for all *i*, $\frac{\alpha_{i}}{\beta_{i}}{\sigma^{\prime }}_{i}^{2}$ equal, or $\beta_{i}\propto \alpha_{i}{\sigma_{i}^{\prime }}^{2}$, makes V[G]=V[F], because then for all *i*, $\alpha_{i}{\sigma^{\prime }}_{i}^{2}=K\beta_{i}$, and substituting in the expressions for V[G],V[F] we have V[G]=V[F]=K, but G≢F. By Cauchy–Schwartz,

(54) $$\begin{array}{c} {\left(\sum_{i=1}^{n}\sqrt{\frac{\alpha_{i}^{3}{\sigma^{\prime }}_{i}^{2}}{\beta_{i}}}\right)}^{2}\leq \left(\sum_{i=1}^{n}\alpha_{i}\right)\left(\sum_{i=1}^{n}\frac{\alpha_{i}^{2}}{\beta_{i}}{\sigma^{\prime }}_{i}^{2}\right), \end{array}$$

with equality only when, for all *i*, $\frac{\alpha_{i}}{\beta_{i}}{\sigma^{\prime }}_{i}^{2}$ are equal.

In the third equality in Equation (53), the solution for all *i*, $\frac{\alpha_{i}^{2}}{\beta_{i}^{2}}{\sigma^{\prime }}_{i}^{2}$, equal, or $\beta_{i}\propto \alpha_{i}\sigma_{i}^{\prime }$, gives the optimal solution for V[G] when $\left\{\alpha_{i}\right\}$ are fixed, Equation (30). By Cauchy–Schwartz,

(55) $$\begin{array}{c} {\left(\sum_{i=1}^{n}\alpha_{i}{\sigma^{\prime }}_{i}\right)}^{2}\leq \left(\sum_{i=1}^{n}\beta_{i}\right)\left(\sum_{i=1}^{n}\frac{\alpha_{i}^{2}}{\beta_{i}}{\sigma^{\prime }}_{i}^{2}\right), \end{array}$$

with equality only when, for all *i*, $\frac{\alpha_{i}^{2}}{\beta_{i}^{2}}{\sigma^{\prime }}_{i}^{2}$ are equal.

We summarize in Table 2 the different possibilities.

## 5. Relationship with $\chi^{2}$ Divergence: A Necessary and Sufficient Condition for V[G]≤V[F]

The variance of an importance sampling estimator can be written in terms of a $\chi^{2}$ divergence if f(x)≥0 [29,30]. As the F estimator can be seen as an importance sampling estimator with pdf $p\left(x\right)=\sum_{i}^{n}p_{i}\left(x\right)$, its variance can be written as

(56) $$\begin{array}{ccc} V[F] & = & \int \frac{f{\left(x\right)}^{2}}{\sum_{i}^{n}\alpha_{i}p_{i}\left(x\right)}dx-\mu^{2}\\ & = & \mu^{2}\int \frac{\frac{f{\left(x\right)}^{2}}{\mu^{2}}}{\sum_{i}^{n}\alpha_{i}p_{i}\left(x\right)}dx-2\mu^{2}\int \frac{f(x)}{\mu }dx+\mu^{2}\int \left(\sum_{i}^{n}\alpha_{i}p_{i}\left(x\right)\right)dx\\ & = & \mu^{2}\int \frac{{\left(\frac{f(x)}{\mu }-\left(\sum_{i}^{n}\alpha_{i}p_{i}\left(x\right)\right)\right)}^{2}}{\sum_{i}^{n}\alpha_{i}p_{i}\left(x\right)}dx\\ & = & \mu^{2}\chi^{2}\left(\frac{f(x)}{\mu },\sum_{i}^{n}\alpha_{i}p_{i}\left(x\right)\right). \end{array}$$

Observe that from the $\chi^{2}$ expression in Equation (56) it is clear that $V\left[F\right]=0\Leftrightarrow f\left(x\right)\propto \sum_{i}^{n}\alpha_{i}p_{i}\left(x\right)$ except possibly in a zero measure set. As a consequence, for all *i*$\mu_{i}^{\prime }=\mu$, and thus V[F]=V[F]=0. As V[F]≤V[F], we have the result

(57) V[F]=0⇔V[F]=0.

A possible generalization of Equation (56) to the G estimator, the randomized version of *G*, is given in Appendix D.

Although neither V[F] nor V[G] can be written as in Equation (56), we can still relate them to $\chi^{2}$ divergence. In general, $\sum_{i}^{n}\alpha_{k}{\sigma^{\prime }}_{k}>0$. Note that, if $\sum_{k}^{n}\alpha_{k}{\sigma^{\prime }}_{k}=0$, then, for all *k*, ${\sigma^{\prime }}_{k}=0$ and V[F]=V[G]=0. Thus both V[G],V[F]=0 or both V[G],V[F]>0. Let us denote $K=\sum_{k=1}^{n}\alpha_{k}{\sigma^{\prime }}_{k}$. Then,

(58) $$\begin{array}{ccc} V[G] & = & \sum_{i=1}^{n}\frac{\alpha_{i}^{2}{\sigma^{\prime }}_{i}^{2}}{\beta_{i}}\\ & = & K^{2}\sum_{i=1}^{n}\frac{\frac{\alpha_{i}^{2}{\sigma^{\prime }}_{i}^{2}}{K^{2}}}{\beta_{i}}+K^{2}\sum_{i=1}^{n}\beta_{i}-K^{2}+K^{2}\sum_{i=1}^{n}2\frac{\alpha_{i}{\sigma^{\prime }}_{i}}{K}-K^{2}\sum_{i=1}^{n}2\frac{\alpha_{i}{\sigma^{\prime }}_{i}}{K}\\ & = & K^{2}\left(\sum_{i=1}^{n}\frac{\frac{\alpha_{i}^{2}{\sigma^{\prime }}_{i}^{2}}{K^{2}}-2\frac{\alpha_{i}{\sigma^{\prime }}_{i}}{K}\beta_{i}+\beta_{i}^{2}}{\beta_{i}}\right)-K^{2}+K^{2}\sum_{i=1}^{n}2\frac{\alpha_{i}{\sigma^{\prime }}_{i}}{K}\\ & = & K^{2}\left(\sum_{i=1}^{n}\frac{\frac{\alpha_{i}^{2}{\sigma^{\prime }}_{i}^{2}}{K^{2}}-2\frac{\alpha_{i}{\sigma^{\prime }}_{i}}{K}\beta_{i}+\beta_{i}^{2}}{\beta_{i}}\right)+K^{2}\\ & = & K^{2}\left(\sum_{i=1}^{n}\frac{{\left(\frac{\alpha_{i}{\sigma^{\prime }}_{i}}{K}-\beta_{i}\right)}^{2}}{\beta_{i}}+1\right)\\ & = & {\left(\sum_{k=1}^{n}\alpha_{k}{\sigma^{\prime }}_{k}\right)}^{2}\left(\chi^{2}\left(\left\{\frac{\alpha_{i}{\sigma^{\prime }}_{i}}{\sum_{k=1}^{n}\alpha_{k}{\sigma^{\prime }}_{k}}\right\},\left\{\beta_{i}\right\}\right)+1\right). \end{array}$$

For ${\left\{\beta_{i}\propto \alpha_{i}\sigma^{\prime }\right\}}_{i=1}^{n}$ the $\chi^{2}$ divergence value is zero, and $V\left[G\right]={\left(\sum_{k=1}^{n}\alpha_{k}{\sigma^{\prime }}_{k}\right)}^{2}$. This is the optimal of V[G] when the ${\left\{\alpha_{i}\right\}}_{i=1}^{n}$ are fixed, as seen in Section 3.2.

Substituting ${\left\{\beta_{i}=\alpha_{i}\right\}}_{i=1}^{n}$ in Equation (58) we obtain

(59) $$\begin{array}{c} V\left[F\right]={\left(\sum_{k=1}^{n}\alpha_{k}{\sigma^{\prime }}_{k}\right)}^{2}\left(\chi^{2}\left(\left\{\frac{\alpha_{i}{\sigma^{\prime }}_{i}}{\sum_{k=1}^{n}\alpha_{k}{\sigma^{\prime }}_{k}}\right\},\left\{\alpha_{i}\right\}\right)+1\right). \end{array}$$

As we have taken $\sum_{k=1}^{n}\alpha_{k}{\sigma^{\prime }}_{k}>0$, we can divide first and last terms of Equations (58) and (59),

(60) $$\begin{array}{c} V\left[G\right]=V\left[F\right]\frac{\chi^{2}\left(\left\{\frac{\alpha_{i}{\sigma^{\prime }}_{i}}{\sum_{k=1}^{n}\alpha_{k}{\sigma^{\prime }}_{k}}\right\},\left\{\beta_{i}\right\}\right)+1}{\chi^{2}\left(\left\{\frac{\alpha_{i}{\sigma^{\prime }}_{i}}{\sum_{k=1}^{n}\alpha_{k}{\sigma^{\prime }}_{k}}\right\},\left\{\alpha_{i}\right\}\right)+1}. \end{array}$$

Hence the following theorem,

**Theorem** **13.** *If V[G],V[F]>0, then*

$$V\left[G\right]\leq V\left[F\right]\Leftrightarrow \chi^{2}\left(\left\{\frac{\alpha_{i}{\sigma^{\prime }}_{i}}{\sum_{k=1}^{n}\alpha_{k}{\sigma^{\prime }}_{k}}\right\},\left\{\beta_{i}\right\}\right)\leq \chi^{2}\left(\left\{\frac{\alpha_{i}{\sigma^{\prime }}_{i}}{\sum_{k=1}^{n}\alpha_{k}{\sigma^{\prime }}_{k}}\right\},\left\{\alpha_{i}\right\}\right).$$

Observe that Theorem 13 does not impose any a priori condition on the $\left\{\alpha_{i}\right\},\left\{\beta_{i}\right\}$ parameters and generalizes Theorem 12. In the same way we can generalize Theorems 7–11 with the following corollaries,

**Corollary** **3.** *If $V\left[G\right],V[G\left(\left\{\beta_{i}=1/n\right\}\right)]>0$, then*

$$V\left[G\right]\leq V\left[G\left(\left\{\beta_{i}=1/n\right\}\right]\right)\Leftrightarrow \chi^{2}\left(\left\{\frac{\alpha_{i}{\sigma^{\prime }}_{i}}{\sum_{k=1}^{n}\alpha_{k}{\sigma^{\prime }}_{k}}\right\},\left\{\beta_{i}\right\}\right)\leq \chi^{2}\left(\left\{\frac{\alpha_{i}{\sigma^{\prime }}_{i}}{\sum_{k=1}^{n}\alpha_{k}{\sigma^{\prime }}_{k}}\right\},\left\{1/n\right\}\right).$$

**Corollary** **4.** *If $V\left[G\left(\left\{\beta_{i}=1/n\right\}\right)\right],V\left[F\right]>0$, then*

$$V\left[G\left(\left\{\beta_{i}=1/n\right\}\right)\right]\leq V\left[F\right]\Leftrightarrow \chi^{2}\left(\left\{\frac{\alpha_{i}{\sigma^{\prime }}_{i}}{\sum_{k=1}^{n}\alpha_{k}{\sigma^{\prime }}_{k}}\right\},\left\{1/n\right\}\right)\leq \chi^{2}\left(\left\{\frac{\alpha_{i}{\sigma^{\prime }}_{i}}{\sum_{k=1}^{n}\alpha_{k}{\sigma^{\prime }}_{k}}\right\},\left\{\alpha_{i}\right\}\right).$$

**Remark** **4.** *Observe that, using the second order $\chi^{2}$ approximation of KL (Kullback–Leibler) distance between two distributions $X_{1},X_{2}$, $\chi^{2}\left(X_{1},X_{2}\right)\approx 2KL\left(X_{1},X_{2}\right)$ [31], Theorem 13 can be written as*

$$\begin{array}{ccc} V[G] & \leq & V\left[F\right]\Leftrightarrow KL\left(\left\{\frac{\alpha_{i}{\sigma^{\prime }}_{i}}{\sum_{k=1}^{n}\alpha_{k}{\sigma^{\prime }}_{k}}\right\},\left\{\beta_{i}\right\}\right)\leq KL\left(\left\{\frac{\alpha_{i}{\sigma^{\prime }}_{i}}{\sum_{k=1}^{n}\alpha_{k}{\sigma^{\prime }}_{k}}\right\},\left\{\alpha_{i}\right\}\right)\\ & \Leftrightarrow & CE\left(\left\{\frac{\alpha_{i}{\sigma^{\prime }}_{i}}{\sum_{k=1}^{n}\alpha_{k}{\sigma^{\prime }}_{k}}\right\},\left\{\beta_{i}\right\}\right)\leq CE\left(\left\{\frac{\alpha_{i}{\sigma^{\prime }}_{i}}{\sum_{k=1}^{n}\alpha_{k}{\sigma^{\prime }}_{k}}\right\},\left\{\alpha_{i}\right\}\right), \end{array}$$

*where cross entropy $CE\left(X_{1},X_{2}\right)=H\left(X_{1}\right)+KL\left(X_{1},X_{2}\right)$, and H stands for entropy.*

The same approximation can be used for Corollaries 3 and 4.

## 6. Numerical Examples

### 6.1. Efficiency Comparison between F and G Estimators

We compare the efficiencies for the *F* estimator and the optimal *G* estimator defined in Theorem 5 with five examples. Table 3 shows the inverse of the efficiencies, $E_{F}^{-1}=V\left[F\right]\cdot Cost\left[F\right]$ and $E_{G}^{-1}=V\left[G\right]\cdot Cost\left[G\right]$, i.e., the product of variance and cost for the *F* estimator and for the optimal *G* estimator, for these possible sets of ${\left\{\alpha_{k}\right\}}_{k=1}^{n}$: (i) equal count of samples, $\left\{\alpha_{k}=1/n\right\}$; (ii) count of samples inversely proportional to the variances of the independent techniques times the cost of sampling the technique, $\left\{\alpha_{k}\propto \frac{1}{c_{k}v_{k}}\right\}$ [8,9]; and the three balance heuristic estimators defined in [28], which are (iii) count of samples inversely proportional to the second moments of the independent techniques times the cost of sampling the technique, $\left\{\alpha_{k}\propto \frac{1}{c_{k}m_{k}^{2}}\right\}$; (iv) count of samples proportional to $\sigma_{k,eq}=\frac{\sigma_{k}^{\prime }\left({\left\{\alpha_{i}=1/n\right\}}_{i=1}^{n}\right)}{n}$, and inversely proportional to the square root of the cost, $\left\{\alpha_{k}\propto \frac{\sigma_{k,eq}}{\sqrt{c_{k}}}\right\}$; and (v) $\left\{\alpha_{k}\propto \frac{M_{k,eq}}{\sqrt{c_{k}}}\right\}$ where $M_{k,eq}=\sqrt{\int \frac{f^{2}\left(x\right)p_{k}\left(x\right)}{{\left(\sum_{i}p_{i}\left(x\right)\right)}^{2}}}$.

Below, we describe the five examples. From the results in Table 3, we can see:

- As expected, Theorems 4 and 5 hold for all the cases.
- Examples 1–4 show a general gain in efficiency, around 20%.
- Example 5 with equal costs shows a gain in efficiency from 2% to around 20%.
- Example 5 with different costs shows big gains in efficiency, in particular for equal count of sampling the efficiency is doubled.

We see in all our examples an increase in efficiency when the estimator *G* is used instead of the estimator *F*, showing in some cases, as in Example 5, a 50% improvement. Theorems 4 and 5 ensure that estimator *G* is always better than estimator *F* if the optimal $\left\{\beta_{i}\right\}$ values are used; however, for equal costs (i.e., comparing only variances), the amount of improvement strongly depends on the example considered, from very small o negligible (see first three rows of Table 4 for the first four examples) to an important one (compare Table 3 for Example 5 with equal costs with the first three rows of Table 4 for Example 5). The values are computed for the results in Table 3 and Table 4 by using numerical approximations obtained with Mathematica software. It would also be possible to select them in an adaptive and iterative way. We defer the investigation of these adaptive schemes for a future work.

#### 6.1.1. Example 1

Suppose we want to solve the integral

(61) $$\mu =\int_{\frac{3}{2\pi }}^{\pi }x\left(x^{2}-\frac{x}{\pi }\right)\sin\left(x\right)dx\approx 10.29$$

by MIS sampling on functions x, $\left(x^{2}-\frac{x}{\pi }\right)$, and sin(x), respectively. We first find the normalization constants: $\int_{\frac{3}{2\pi }}^{\pi }xdx=4.82082$, $\int_{\frac{3}{2\pi }}^{\pi }\left(x^{2}-\frac{x}{\pi }\right)dx=8.76463$, $\int_{\frac{3}{2\pi }}^{\pi }\sin\left(x\right)dx=1.88816$. The costs for sampling the techniques are (1; 6.24; 3.28). 

#### 6.1.2. Example 2

Let us solve the integral

(62) $$\mu =\int_{\frac{3}{2\pi }}^{\pi }\left(x^{2}-\frac{x}{\pi }\right)\sin^{2}\left(x\right)dx\approx 3.60$$

using the same functions x, $\left(x^{2}-\frac{x}{\pi }\right)$, and sin(x) as before.

#### 6.1.3. Example 3

As the third example, let us solve the integral

(63) $$\mu =\int_{\frac{3}{2\pi }}^{\pi }x+\left(x^{2}-\frac{x}{\pi }\right)+\sin\left(x\right)dx\approx 15.47\;$$

using the same functions as before. The optimal α values for this example, with zero variance, are $\frac{1}{4.82082+8.76463+1.88816}\left(4.82082,8.76463,1.88816\right)$.

#### 6.1.4. Example 4

As the fourth example, consider the integral of the sum of the three pdfs

(64) $$\begin{array}{cc} \;\mu & =\int_{\frac{3}{2\pi }}^{\pi }30\frac{x}{4.82082}+30\frac{\left(x^{2}-\frac{x}{\pi }\right)}{8.76463}+40\frac{\sin(x)}{1.88816}dx\\ \; & \approx 100. \end{array}$$

In this case we know again the optimal (zero variance) α values: (0.3,0.3,0.4). The difference with the previous example is that this case should be most favorable to equal count of samples.

#### 6.1.5. Example 5

As the last example, consider solving the following integral

(65) $$\begin{array}{c} \;\mu =\int_{0.01}^{\pi /2}\left(\sqrt{x}+\sin x\right)dx\approx 2.31175. \end{array}$$

by MIS sampling on functions 2−x, and $\sin^{2}\left(x\right)$.

### 6.2. Variances of Examples 1–5 for Some Notable Cases

In Table 4 we present some notable cases for the variance of Examples 1–5. Observe that here we do not consider the cost of sampling. The values are computed for the results in Table 4 with Mathematica software. We have

- In first, second and third row we present the optimal values of V[F]≥V[F]≥V[G], respectively. In the first example those values are equal up to the fourth decimal position. In the second example they are equal up to the second decimal position, in the third and fourth examples they are equal, and in the fifth example the gain of V[G] with respect to V[F] is more than 30%.
- In the fourth row we present the values of V[F(1/n)], which are compared against several strategies and heuristics in the following rows
- The values in the fifth row for V[G], that correspond to Equation (30) (or the left-hand side of Equation (37) with all costs equal to 1), although being better than the values for V[F] in the fourth row, as expected, do not give any significant improvement. As we have seen in Table 3 the improvements for this case come rather from efficiency.
- The values in the sixth row for V[G], that correspond to the solution of Equation (40), this is, the optimal $\left\{\alpha_{i}\right\}$ for $\left\{\beta_{i}=1/n\right\}$, are smaller than the variances of V[F] for equal sampling rate, fourth row, and much smaller in some of the examples, with variances equal (as in Examples 3 and 4), or less, as in Example 5, than the optimal V[F].
- Although in general there is no $\left\{\alpha_{i}\right\}$ solution for all $\mu_{i}^{\prime }$ equal, for instance the numerical approximation obtained in Example 1 is $\mu_{1}^{\prime }=10.2624$, $\mu_{2}^{\prime }=\mu_{3}^{\prime }=10.2876$, the variances obtained, in the seventh row of Table 4, are for all the examples very near to the optimal value of V[F]. Observe that if these $\left\{\alpha_{i}^{⋆}\right\}=arg\;\forall i,\mu_{i}^{\prime }\;equal$ values exist, then they are the optimal $\left\{\alpha_{i}\right\}$ values for $V[G\left(\beta_{i}=\alpha_{i}^{⋆}\right)]$, i.e., for the sampling rates $\left\{\beta_{i}=\alpha_{i}^{⋆}\right\}$ the *G* estimator can not improve on the *F* estimator.
- In row 8 we show the *G* estimator corresponding $\alpha_{i}\sigma_{i}^{\prime }\approx equal$, with $\beta_{i}=1/n$. It improves on F(1/n) for all examples except the third one, where it scores closely, beating estimators in rows 9 and 10 except for Example 3. It is indeed a theoretical estimator as there is no easy way to solve this equation. However, it can be approximated by the heuristic in line 11, see below. Observe that if $\left\{\alpha_{i}^{⋆}\right\}$ are the solutions of for all *i*, $\alpha_{i}\sigma_{i}^{\prime }$ equal, then the optimal $\left\{\beta_{i}\right\}$ for $G(\left\{\alpha_{i}^{⋆}\right\},\left\{\beta_{i}\right\})$ are $\left\{\beta_{i}=1/n\right\}$.
- The results in row 9 correspond to the estimator defined in [19], $\alpha_{i}\propto m_{i}^{2}/v_{i}$, where $m_{i}^{2}=v_{i}+\mu^{2}$ are the second moments of the independent techniques, and $\beta_{i}=1/n$. The results are slightly better than F(1/n) for Examples 1 and 2, much better for Example 3, much worse for Example 4 and slightly worse for Example 5. Observe that the $m_{i},v_{i}$ values can be easily approximated using a first batch of samples in a Monte Carlo integration, as it was already performed in [7,9,10].
- In row 10 we introduce the estimator corresponding to $\alpha_{i}\propto 1/v_{i}$, and $\beta_{i}=1/n$, which gives better results than F(1/n) for Examples 1 and 2, much better for Example 3, worse for Example 5, and much worse for Example 4. This estimator improves on the one in [19] except for Example 4. Observe that these $\alpha_{i}$ weights correspond to the optimal weights in the linear combination of estimators when the sampling rates ($\beta_{i}$) are fixed, Equation (13) of [9].
- In row 11 we approximate the estimator in row 8 by using $\left\{\sigma_{i,1/n}^{\prime }\right\}$, which corresponds to $\left\{\alpha_{i}=1/n\right\}$, to approximate $\left\{\sigma_{i}^{\prime }\right\}$. Observe that $\left\{\sigma_{i,1/n}^{\prime }\right\}$ values can be easily obtained with a first batch of samples in a Monte Carlo integration. In all cases, except in Example 3, we improve on F(1/n). The big error on Example 4 of the estimator in lines 9 and 10 is controlled.
- In row 12 we modulate the estimator in row 11 with the $\mu_{i}^{\prime }$ values. Observe that now for all the examples the variance is less than for F(1/n).
- The results in row 13 show that, even if $F\left(\left\{\alpha_{i}\right\}\right)\leq F\left(\left\{1/n\right\}\right)$, we can not guarantee that $G\left(\left\{\alpha_{i}\right\},\left\{\beta_{i}=1/n\right\}\right)\leq F\left(\left\{1/n\right\}\right)$.

From the results in Table 4 row 6, we can conclude that there can be room for improvement on F(1/n) estimator using a *G* estimator, with a suitable selection of $\alpha_{i}$ parameters. Heuristics as in rows 9 and 10 can give much improvement in some cases at the cost of no improvement at all and even a huge variance in other cases, thus they are not robust heuristics. More sophisticated heuristics as in rows 11 and 12, based on the theoretically justified heuristic in row 8, show a robust behavior.

A very promising estimator is the one in row 7, for all *i* all $\mu_{i}^{\prime }$ equal (to μ), in all five examples the variance is very near the optimal V[F]. Observe that this estimator has a strong theoretical justification, derived from the study of *G* estimator. The $\left\{\alpha_{i}\right\}$ parameters that approximate for all *i*, $\mu_{i}^{\prime }=\mu$ could be obtained in an adaptive way; however, a practical way to obtain this estimator should be devised.

## 7. Conclusions

In this paper, we have proposed a multiple importance sampling estimator that combines samples simulated from different techniques. The novel estimator generalizes the Monte Carlo balance heuristic estimator, widely used in the literature of signal processing, computational statistics, and computer graphics. In particular, this estimator relaxes the connection between the coefficients that select the number of samples per proposal, and the samples that appear in the mixture of techniques at the denominator of the importance weight. This flexibility shows a relevant improvement in terms of variance in the combined estimator with regard to the balance heuristic estimator (which is included as a particular case in the novel estimator). We have studied the optimal choice of the free coefficients in such a way that the variance of the resulting estimator is minimized. In addition, numerical results have shown that the significant gap in terms of variance between both estimators justifies the use of the novel estimator whenever possible. In the particular, but much used case, of equal sampling, our new estimator shows a big potential for improvement. Future work may include the application of this variance-reduction technique in the context of adaptive importance sampling [15] or within MCMC-based methods that include re-weighting [32].

## Figures and Tables

**Table 1:** Naming convention for the multiple importance sampling estimators in this paper. We drop the superindex${}^{1}$ from primary estimators when not strictly necessary.

*Z*	Generic deterministic (multi-sample) MIS estimator
$Z^{1}$	Generic deterministic (multi-sample) MIS estimator normalized to one sample
Z	Generic randomized (one-sample) MIS estimator
$Z^{1}$	Generic randomized (one-sample) MIS estimator, for number of samples equal to 1
*F*	Balance heuristic multi-sample MIS estimator
$F^{1}$	Balance heuristic multi-sample MIS estimator normalized to one sample
F	Balance heuristic one-sample MIS estimator
$F^{1}$	Balance heuristic one-sample MIS estimator, for number of samples equal to 1
*G*	Generalized balance heuristic multi-sample MIS estimator
$G^{1}$	Generalized balance heuristic multi-sample MIS estimator normalized to one sample
G	Generalized balance heuristic one-sample MIS estimator

**Table 2:** Singular parameter values. ${\left\{\alpha_{i}^{⋆}\right\}}_{i=1}^{n},{\left\{\beta_{i}^{⋆}\right\}}_{i=1}^{n}$ are solutions of the equations in first column and same row.

for all *i*, ${\sigma_{i}^{\prime }}^{2}+{\mu^{\prime }}_{i}^{2}$ equal	$V[F\left(\left\{\alpha_{i}^{⋆}\right\}\right)]$ global minimum
for all *i*, ${\sigma_{i}^{\prime }}^{2}$ equal and ${\mu^{\prime }}_{i}$ equal	$V\left[F\left(\left\{\alpha_{i}^{⋆}\right\}\right)\right]=V\left[F(\left\{\alpha_{i}^{⋆}\right\})\right]=V\left[G(\left\{\alpha_{i}^{⋆}\right\},\left\{\beta_{i}\right\}=\left\{\alpha_{i}^{⋆}\right\})\right]$ global minimum, F≡G, but F≢F
for all *i*, $\mu_{i}^{\prime }$ equal	$V\left[F\left(\left\{\alpha_{i}^{⋆}\right\}\right)\right]=V\left[F(\left\{\alpha_{i}^{⋆}\right\})\right]$; optimal of $V[G\left(\left\{\alpha_{i}\right\},\left\{\beta_{i}\right\}=\left\{\alpha_{i}^{⋆}\right\}\right)]$ is for $\left\{\alpha_{i}\right\}=\left\{\alpha_{i}^{⋆}\right\}$
for all *i*, $\sigma_{i}^{\prime }$ equal	optimal of $V[G\left(\left\{\alpha_{i}^{⋆}\right\},\left\{\beta_{i}\right\}\right)]$ is for $\left\{\beta_{i}\right\}=\left\{\alpha_{i}^{⋆}\right\}$, F≡G
for all *i*, $\frac{\alpha_{i}}{\beta_{i}}{\sigma^{\prime }}_{i}^{2}$ equal, or $\beta_{i}\propto \alpha_{i}{\sigma_{i}^{\prime }}^{2}$	$V\left[G\left(\left\{\alpha_{i}^{⋆}\right\},\left\{\beta_{i}^{⋆}\right\}\right)\right]=V\left[F\left(\left\{\alpha_{i}^{⋆}\right\}\right)\right]$
for all *i*, $\frac{\alpha_{i}^{2}}{\beta_{i}^{2}}{\sigma^{\prime }}_{i}^{2}$, equal, or $\beta_{i}\propto \alpha_{i}\sigma_{i}^{\prime }$	optimal of $V[G\left(\left\{\alpha_{i}\right\},\left\{\beta_{i}\right\}\right)]$ when $\left\{\alpha_{i}\right\}$ are fixed, $\forall \left\{\alpha_{i}\right\},V\left[G\left(\left\{\alpha_{i}\right\},\left\{\beta_{i}^{⋆}\right\}\right)\right]\leq V\left[F\left(\left\{\alpha_{i}\right\}\right)\right]$

**Table 3:** We show the metric $E_{F}^{-1}=V\left[F\right]\cdot Cost\left[F\right]$ and $E_{G}^{-1}=V\left[G\right]\cdot Cost\left[G\right]$, i.e., the inverse of efficiency or product of variance and cost for the *F* estimator and for the optimal *G* estimator, using for both the same $\left\{\alpha_{k}\right\}$ values. The optimal efficiencies of *G* estimator, for each set of $\left\{\alpha_{k}\right\}$ values, are computed using the left-hand side of Equation (37), while the efficiencies of *F* estimator use the right-hand side of Equation (37). We consider for the five numerical examples using an equal count of samples, count inversely proportional to the variances of independent estimators [8,9], and for the three estimators defined in [28]. The sampling costs are (1, 6.24, 3.28). In Example 5, we present the case with equal costs (1, 1), and different costs (1, 5).

	Ex. 1		Ex. 2		Ex. 3		Ex. 4		Ex. 5		Ex. 5	
									costs = (1,1)	costs = (1,1)	costs = (1,5)	costs = (1,5)
**Estimator**	**F**	**G**	**F**	**G**	**F**	**G**	**F**	**G**	**F**	**G**	**F**	**G**
$\alpha_{k}\propto \frac{1}{n}$	102.26	89.40	17.24	15.44	37.47	31.80	98.68	83.78	0.28	0.23	0.83	0.40
$\alpha_{k}\;\propto \;\frac{1}{c_{k}v_{k}}$ [8]	49.53	41.29	9.28	8.10	4.03	3.85	300.12	294.85	0.31	0.26	2.76	2.33
$\alpha_{k}\;\propto \;\frac{1}{c_{k}m_{k}^{2}}$ [28]	54.36	46.2	9.82	8.49	3.12	2.49	534.37	449.33	0.20	0.15	1.51	1.00
$\alpha_{k}\;\propto \;\frac{\sigma_{k,eq}}{\sqrt{c_{k}}}$ [28]	81.43	69.88	13.54	11.67	28.68	23.17	91.01	73.54	1.00	0.98	2.90	2.50
$\alpha_{k}\;\propto \;\frac{M_{k,eq}}{\sqrt{c_{k}}}$ [28]	79.73	67.77	13.08	11.35	25.74	20.76	31.77	25.90	0.29	0.24	2.72	2.28

**Table 4:** Variances of Examples 1–5 for some notable cases. The variables ${\mu^{\prime }}_{i,1/n},{\sigma^{\prime }}_{i,1/n}$ correspond to $\left\{\alpha_{i}=\frac{1}{n}\right\}$. The variables $m_{i}^{2},v_{i}$ are the second moment and variance of independent technique *i*, $v_{i}=m_{i}^{2}-\mu^{2}$.

	Estimator	Example 1	Example 2	Example 3	Example 4	Example 5
1	$Optimal_{\alpha }\;V\left[F\right]$	22.7122	4.1949	0	0	0.0910
2	$Optimal_{\alpha }V\left[F\right]$	22.7122	4.1944	0	0	0.0903
3	$Optimal_{\alpha ,\beta }V\left[G\right]$	22.7122	4.1932	0	0	0.0601
4	$V[F\left(\alpha_{i}=1/n\right)]$	29.1634	4.9175	10.6877	28.1431	0.2771
5	$Optimal_{\beta }V\left[G\left(\alpha_{i}=1/n\right)\right]$	29.0908	4.9069	10.6125	27.9412	0.2264
6	$Optimal_{\alpha }V\left[G\left(\beta_{i}=1/n\right)\right]$	27.1603	4.7265	0	0	0.0653
7	$V[F\left(\alpha_{i}=arg\;\forall i,\mu_{i}^{\prime }\approx \mu \right)]$	22.8216	4.1980	0	0	0.0926
8	$V[G\left(\alpha_{i}=arg\;\forall i,\alpha_{i}\sigma_{i}^{\prime }\approx \mathrm{equal},\beta_{i}=1/n\right)]$	28.4089	4.8313	12.4705	11.2072	0.0734
9	$V[G\left(\alpha_{i}\propto \frac{m_{i}^{2}}{v_{i}},\beta_{i}=1/n\right)]$,^19^]	28.0224	4.8513	3.7955	172.192	0.2832
10	$V[G\left(\alpha_{i}\propto \frac{1}{v_{i}},\beta_{i}=1/n\right)]$	27.4126	4.7897	0.5466	1256.48	0.2943
11	$V[G\left(\alpha_{i}\propto \frac{1}{\sigma_{i,1/n}^{\prime }},\beta_{i}=1/n\right)]$	28.3977	4.8291	11.95	13.7926	0.0724
12	$V[G\left(\alpha_{i}\propto \frac{{\mu^{\prime }}_{i,1/n}}{{\sigma^{\prime }}_{i,1/n}},\beta_{i}=1/n\right))]$	27.4426	4.7373	7.8615	6.7895	0.1112
13	$V[G\left(\alpha_{i}=arg\;\forall i,\mu_{i}^{\prime }\approx \mu ,\beta_{i}=1/n\right)]$	41.8791	8.8798	0.0014	0	0.0739

## Appendix A. Difference between the Variances of Deterministic and Randomized Multiple Importance Sampling Estimators

**Proof of Equation (9).** The difference between the variances of the deterministic multiple importance sampling estimator, *Z*, and the randomized one, Z, is given by [17] (we normalize here to one sample)

(A1) $$\begin{array}{ccc} V\left[Z^{1}\right]-V\left[Z^{1}\right] & = & \sum_{i}\alpha_{i}{\mu^{\prime }}_{i}^{2}-\mu^{2}\\ & = & \sum_{i}\alpha_{i}{\mu^{\prime }}_{i}^{2}-{\left(\sum_{i}\alpha_{i}{\mu^{\prime }}_{i}\right)}^{2}, \end{array}$$

which is positive, as by Cauchy-Schwartz

(A2) $$\begin{array}{c} {\left(\sum_{i}\alpha_{i}{\mu^{\prime }}_{i}\right)}^{2}\leq \left(\sum_{i}\alpha_{i}\right)\left(\sum_{i}\alpha_{i}{\mu^{\prime }}_{i}^{2}\right), \end{array}$$

and equality only happens (apart from the case when both $V\left[Z^{1}\right],V\left[Z^{1}\right]$ are zero) when for all *i*, $\alpha_{i}\propto \alpha_{i}{\mu^{\prime }}_{i}^{2}$, i.e., all ${\mu^{\prime }}_{i}$ are equal. □

**Proof of Equation (10).** Observe that if f(x)≥0 then for all *i*$\mu_{i}^{\prime }\geq 0$, and we have

(A3) $$\begin{array}{c} {\left(\sum_{i}{\mu^{\prime }}_{i}\right)}^{2}\geq \sum_{i}{\mu^{\prime }}_{i}^{2}, \end{array}$$

and thus

(A4) $$\begin{array}{c} \mu^{2}={\left(\sum_{i}\frac{1}{n}{\mu^{\prime }}_{i}\right)}^{2}\geq \frac{1}{n}\left(\sum_{i}\frac{1}{n}{\mu^{\prime }}_{i}^{2}\right), \end{array}$$

where equality would be approached when there is an index *k* such that for all i≠k, $\mu_{k}^{\prime }>>\mu_{i}^{\prime }$. Thus when $\alpha_{i}=1/n$,

(A5) $$\begin{array}{c} V\left[Z^{1}\right]-V\left[Z^{1}\right]\leq \left(n-1\right)\mu^{2}. \end{array}$$

□

In general, the more different the $\mu_{i}^{\prime }$ are, the higher the difference between the variances.

## Appendix B. Proof of Theorem 2: Optimal Variance of *F*

**Proof.** The ${\left\{\alpha_{i}\right\}}_{i=1}^{n}$ values for the optimal variance of *F* estimator can be obtained using Lagrange multipliers with the target function

$$\Lambda \left({\left\{\alpha_{i}\right\}}_{i=1}^{n},\lambda \right)=\sum_{i=1}^{n}\alpha_{i}{\sigma^{\prime }}_{i}^{2}+\lambda \left(\sum_{i=1}^{n}\alpha_{i}=1\right).$$

Taking partial derivatives with respect to $\alpha_{j}$,

(A6) $$\begin{array}{ccc} \frac{\partial \Lambda ({\left\{\alpha_{i}\right\}}_{i=1}^{n},\lambda )}{\partial_{\alpha_{j}}} & = & \frac{\partial \left(\sum_{i=1}^{n}\alpha_{i}{\sigma^{\prime }}_{i}^{2}\right)}{\partial_{\alpha_{j}}}+\frac{\partial \left(\lambda \left(\sum_{i=1}^{n}\alpha_{i}-1\right)\right)}{\partial_{\alpha_{j}}}\\ & = & \sum_{i=1}^{n}\frac{\partial \left(\alpha_{i}{\sigma^{\prime }}_{i}^{2}\right)}{\partial_{\alpha_{j}}}+\lambda =0. \end{array}$$

The partial derivatives are equal to

(A7) $$\begin{array}{c} \frac{\partial \left(\alpha_{i}{\sigma^{\prime }}_{i}^{2}\right)}{\partial_{\alpha_{j}}}=\chi_{i}\left(j\right){\sigma^{\prime }}_{j}^{2}+\alpha_{i}\frac{\partial \left({\sigma^{\prime }}_{i}^{2}\right)}{\partial_{\alpha_{j}}}, \end{array}$$

where $\chi_{i}\left(j\right)$ is the characteristic function, $\chi_{i}\left(j\right)=1$ if i=j and 0 if i≠j.

(A8) $$\begin{array}{ccc} \frac{\partial \left({\sigma^{\prime }}_{i}^{2}\right)}{\partial_{\alpha_{j}}} & = & \frac{\partial \left(\int \frac{f^{2}\left(x\right)p_{i}\left(x\right)}{{\left(\sum_{k=1}^{n}\alpha_{k}p_{k}\left(x\right)\right)}^{2}}dx-{\left(\mu_{i}^{\prime }\right)}^{2}\right)}{\partial_{\alpha_{j}}}\\ & = & \frac{\partial \left(\int \frac{f^{2}\left(x\right)p_{i}\left(x\right)}{{\left(\sum_{k=1}^{n}\alpha_{k}p_{k}\left(x\right)\right)}^{2}}dx\right)}{\partial_{\alpha_{j}}}-2\mu_{i}^{\prime }\frac{\partial \mu_{i}^{\prime }}{\partial_{\alpha_{j}}}\\ & = & -2\int \frac{f^{2}\left(x\right)p_{i}\left(x\right)p_{j}\left(x\right)}{{\left(\sum_{k=1}^{n}\alpha_{k}p_{k}\left(x\right)\right)}^{3}}dx-2\mu_{i}^{\prime }\frac{\partial \mu_{i}^{\prime }}{\partial_{\alpha_{j}}}. \end{array}$$

Since we can write

(A9) $$\begin{array}{ccc} \frac{\partial \mu_{i}^{\prime }}{\partial_{\alpha_{j}}} & = & \frac{\partial \left(\int \frac{f\left(x\right)p_{i}\left(x\right)}{\sum_{k=1}^{n}\alpha_{k}p_{k}\left(x\right)}dx\right)}{\partial_{\alpha_{j}}}\\ & = & -\int \frac{f\left(x\right)p_{i}\left(x\right)p_{j}\left(x\right)}{{\left(\sum_{k=1}^{n}\alpha_{k}p_{k}\left(x\right)\right)}^{2}}dx, \end{array}$$

thus Equation (A8) reads

(A10) $$\begin{array}{ccc} \frac{\partial \left({\sigma^{\prime }}_{i}^{2}\right)}{\partial_{\alpha_{j}}} & = & -2\int \frac{f^{2}\left(x\right)p_{i}\left(x\right)p_{j}\left(x\right)}{{\left(\sum_{k=1}^{n}\alpha_{k}p_{k}\left(x\right)\right)}^{3}}dx\\ & + & 2\mu_{i}^{\prime }\int \frac{f\left(x\right)p_{i}\left(x\right)p_{j}\left(x\right)}{{\left(\sum_{k=1}^{n}\alpha_{k}p_{k}\left(x\right)\right)}^{2}}dx. \end{array}$$

Then,

(A11) $$\begin{array}{ccc} & & \lambda =-\sum_{i=1}^{n}\frac{\partial \left(\alpha_{i}{\sigma^{\prime }}_{i}^{2}\right)}{\partial_{\alpha_{j}}}=-{\sigma^{\prime }}_{j}^{2}+2\sum_{i=1}^{n}\alpha_{i}\times \\ & & \left(\int \frac{f^{2}\left(x\right)p_{i}\left(x\right)p_{j}\left(x\right)}{{\left(\sum_{k=1}^{n}\alpha_{k}p_{k}\left(x\right)\right)}^{3}}dx-\mu_{i}^{\prime }\int \frac{f\left(x\right)p_{i}\left(x\right)p_{j}\left(x\right)}{{\left(\sum_{k=1}^{n}\alpha_{k}p_{k}\left(x\right)\right)}^{2}}dx\right)\\ & = & -{\sigma^{\prime }}_{j}^{2}+2\int \frac{f^{2}\left(x\right)p_{j}\left(x\right)\left(\sum_{i=1}^{n}\alpha_{i}p_{i}\left(x\right)\right)}{{\left(\sum_{k=1}^{n}\alpha_{k}p_{k}\left(x\right)\right)}^{3}}dx\\ & - & 2\sum_{i=1}^{n}\alpha_{i}\mu_{i}^{\prime }\int \frac{f\left(x\right)p_{i}\left(x\right)p_{j}\left(x\right)}{{\left(\sum_{k=1}^{n}\alpha_{k}p_{k}\left(x\right)\right)}^{2}}dx\\ & = & -{\sigma^{\prime }}_{j}^{2}+2\left({\sigma^{\prime }}_{j}^{2}+{\mu_{j}^{\prime }}^{2}\right)-2\sum_{i=1}^{n}\alpha_{i}\mu_{i}^{\prime }\int \frac{f\left(x\right)p_{i}\left(x\right)p_{j}\left(x\right)}{{\left(\sum_{k=1}^{n}\alpha_{k}p_{k}\left(x\right)\right)}^{2}}dx\\ & = & {\sigma^{\prime }}_{j}^{2}+2{\mu_{j}^{\prime }}^{2}-2\sum_{i=1}^{n}\alpha_{i}\mu_{i}^{\prime }\int \frac{f\left(x\right)p_{i}\left(x\right)p_{j}\left(x\right)}{{\left(\sum_{k=1}^{n}\alpha_{k}p_{k}\left(x\right)\right)}^{2}}dx \end{array}$$

This is, for all *j*, the following values have to be equal

(A12) $$\begin{array}{c} {\sigma^{\prime }}_{j}^{2}+2{\mu_{j}^{\prime }}^{2}-2\sum_{i=1}^{n}\alpha_{i}\mu_{i}^{\prime }\int \frac{f\left(x\right)p_{i}\left(x\right)p_{j}\left(x\right)}{{\left(\sum_{k=1}^{n}\alpha_{k}p_{k}\left(x\right)\right)}^{2}}dx \end{array}$$

Multiplying by $\alpha_{j}$ and adding over all *j* in Equation (A11),

(A13) $$\begin{array}{ccc} \lambda & = & \sum_{j=1}^{n}\alpha_{j}\lambda =\sum_{j=1}^{n}\alpha_{j}{\sigma^{\prime }}_{j}^{2}+2\sum_{j=1}^{n}\alpha_{j}{\mu_{j}^{\prime }}^{2}\\ & - & 2\sum_{i=1}^{n}\alpha_{i}\mu_{i}^{\prime }\int \frac{f\left(x\right)p_{i}\left(x\right)\left(\sum_{j=1}^{n}\alpha_{j}p_{j}\left(x\right)\right)}{{\left(\sum_{k=1}^{n}\alpha_{k}p_{k}\left(x\right)\right)}^{2}}dx\\ & = & \sum_{j=1}^{n}\alpha_{j}{\sigma^{\prime }}_{j}^{2}+2\sum_{j=1}^{n}\alpha_{j}{\mu_{j}^{\prime }}^{2}-2\sum_{i=1}^{n}\alpha_{i}{\mu_{i}^{\prime }}^{2}\\ & = & \sum_{j=1}^{n}\alpha_{j}{\sigma^{\prime }}_{j}^{2} \end{array}$$

which is the optimal variance of estimator *F*. Equation (A12) becomes, for all *j*

(A14) $$\begin{array}{c} \sum_{j=1}^{n}\alpha_{j}{\sigma^{\prime }}_{j}^{2}={\sigma^{\prime }}_{j}^{2}+2{\mu_{j}^{\prime }}^{2}-2\sum_{i=1}^{n}\alpha_{i}\mu_{i}^{\prime }\int \frac{f\left(x\right)p_{i}\left(x\right)p_{j}\left(x\right)}{{\left(\sum_{k=1}^{n}\alpha_{k}p_{k}\left(x\right)\right)}^{2}}dx. \end{array}$$

Observe that all derivatives in (A6) are negative for the optimal $\left\{\alpha_{i}^{⋆}\right\}$ values and equal to $-\sum_{j=1}^{n}\alpha_{j}^{⋆}{\sigma^{{}^{\prime }⋆}}_{j}^{2}$. □

## Appendix C. Derivation of Case 3

**Proof of Theorem 6.** We have to optimize the target function

$$\Lambda \left({\left\{\alpha_{i}\right\}}_{i=1}^{n},\lambda \right)=\sum_{i=1}^{n}\frac{\alpha_{i}^{2}{\sigma^{\prime }}_{i}^{2}}{\beta_{i}}+\lambda \left(\sum_{i=1}^{n}\alpha_{i}-1\right).$$

Taking partial derivatives with respect to $\alpha_{i}$, as the $\beta_{i}$ values are constant,

(A15) $$\begin{array}{c} \frac{\partial \Lambda ({\left\{\alpha_{i}\right\}}_{i=1}^{n},\lambda )}{\partial_{\alpha_{j}}}=\sum_{i=1}^{n}\frac{1}{\beta_{i}}\frac{\partial \left(\alpha_{i}^{2}{\sigma^{\prime }}_{i}^{2}\right)}{\partial_{\alpha_{j}}}+\lambda =0. \end{array}$$

The partial derivatives are equal to

(A16) $$\begin{array}{c} \frac{\partial \left(\alpha_{i}^{2}{\sigma^{\prime }}_{i}^{2}\right)}{\partial_{\alpha_{j}}}=2\alpha_{j}\chi_{i}\left(j\right){\sigma^{\prime }}_{j}^{2}+\alpha_{i}^{2}\frac{\partial \left({\sigma^{\prime }}_{i}^{2}\right)}{\partial_{\alpha_{j}}}, \end{array}$$

where $\chi_{i}\left(j\right)$ is the characteristic function. Using the result in Equation (A10), we obtain

(A17) $$\begin{array}{ccc} & & \sum_{i=1}^{n}\frac{1}{\beta_{i}}\frac{\partial \left(\alpha_{i}^{2}{\sigma^{\prime }}_{i}^{2}\right)}{\partial_{\alpha_{j}}}=2\frac{\alpha_{j}{\sigma^{\prime }}_{j}^{2}}{\beta_{j}}-2\sum_{i=1}^{n}\frac{\alpha_{i}^{2}}{\beta_{i}}\\ & \times & \left(\int \frac{f^{2}\left(x\right)p_{i}\left(x\right)p_{j}\left(x\right)}{{\left(\sum_{k=1}^{n}\alpha_{k}p_{k}\left(x\right)\right)}^{3}}dx-\mu_{i}^{\prime }\int \frac{f\left(x\right)p_{i}\left(x\right)p_{j}\left(x\right)}{{\left(\sum_{k=1}^{n}\alpha_{k}p_{k}\left(x\right)\right)}^{2}}dx\right)\\ & = & -\lambda . \end{array}$$

In Equation (A17), we multiply by $\alpha_{j}$, and add over all indexes *j*, obtaining

(A18) $$\begin{array}{ccc} \lambda & = & -2\sum_{j=1}^{n}\frac{\alpha_{j}^{2}{\sigma^{\prime }}_{j}^{2}}{\beta_{j}}+2\sum_{i=1}^{n}\frac{\alpha_{i}^{2}}{\beta_{i}}\\ & \times & (\int \frac{f^{2}\left(x\right)p_{i}\left(x\right)\left(\sum_{j=1}^{n}\alpha_{j}p_{j}\left(x\right)\right)}{{\left(\sum_{k=1}^{n}\alpha_{k}p_{k}\left(x\right)\right)}^{3}}dx\\ & - & \mu_{i}^{\prime }\int \frac{f\left(x\right)p_{i}\left(x\right)\left(\sum_{j=1}^{n}\alpha_{j}p_{j}\left(x\right)\right)}{{\left(\sum_{k=1}^{n}\alpha_{k}p_{k}\left(x\right)\right)}^{2}}dx)\\ & = & -2\sum_{j=1}^{n}\frac{\alpha_{j}^{2}{\sigma^{\prime }}_{j}^{2}}{\beta_{j}}+2\sum_{i=1}^{n}\frac{\alpha_{i}^{2}}{\beta_{i}}\\ & \times & \left(\int \frac{f^{2}\left(x\right)p_{i}\left(x\right)}{{\left(\sum_{k=1}^{n}\alpha_{k}p_{k}\left(x\right)\right)}^{2}}dx-\mu_{i}^{\prime }\int \frac{f\left(x\right)p_{i}\left(x\right)}{\left(\sum_{k=1}^{n}\alpha_{k}p_{k}\left(x\right)\right)}dx\right)\\ & = & -2\sum_{j=1}^{n}\frac{\alpha_{j}^{2}{\sigma^{\prime }}_{j}^{2}}{\beta_{j}}+2\sum_{i=1}^{n}\frac{\alpha_{i}^{2}{\sigma^{\prime }}_{i}^{2}}{\beta_{i}}=0. \end{array}$$

We remind that $\sum_{j=1}^{n}\alpha_{j}=1$ which disappears in the left-hand side and the second term of the right-hand side equation. From Equation (A17), the optimal ${\left\{\alpha_{j}^{⋆}\right\}}_{j=1}^{n}$ obey

(A19) $$\begin{array}{ccc} & & \frac{\alpha_{j}^{⋆}{\sigma^{\prime }}_{j}^{2}}{\beta_{j}}=\sum_{i=1}^{n}\frac{{\alpha^{⋆}}_{i}^{2}}{\beta_{i}}\\ & \times & \left(\int \frac{f^{2}\left(x\right)p_{i}\left(x\right)p_{j}\left(x\right)}{{\left(\sum_{k=1}^{n}\alpha_{k}^{⋆}p_{k}\left(x\right)\right)}^{3}}dx-\mu_{i}^{\prime }\int \frac{f\left(x\right)p_{i}\left(x\right)p_{j}\left(x\right)}{{\left(\sum_{k=1}^{n}\alpha_{k}^{⋆}p_{k}\left(x\right)\right)}^{2}}dx\right). \end{array}$$

□

**Proof of Equation (39).** For the optimal solution to be, for all *i*, $\alpha_{i}^{⋆}=\beta_{i}$ then

$$\begin{array}{ccc} {\sigma^{\prime }}_{j}^{2} & = & \int \frac{f^{2}\left(x\right)\left(\sum_{i=1}^{n}\alpha_{i}^{⋆}p_{i}\left(x\right)\right)p_{j}\left(x\right)}{{\left(\sum_{k=1}^{n}\alpha_{k}^{⋆}p_{k}\left(x\right)\right)}^{3}}dx\\ & - & \sum_{i=1}^{n}\alpha_{i}^{⋆}\mu_{i}^{\prime }\int \frac{f\left(x\right)p_{i}\left(x\right)p_{j}\left(x\right)}{{\left(\sum_{k=1}^{n}\alpha_{k}^{⋆}p_{k}\left(x\right)\right)}^{2}}dx\\ & = & {\sigma^{\prime }}_{j}^{2}+{\mu^{\prime }}_{j}^{2}-\sum_{i=1}^{n}\alpha_{i}^{⋆}\mu_{i}^{\prime }\int \frac{f\left(x\right)p_{i}\left(x\right)p_{j}\left(x\right)}{{\left(\sum_{k=1}^{n}\alpha_{k}^{⋆}p_{k}\left(x\right)\right)}^{2}}dx, \end{array}$$

and thus the following equation has to hold for all *j*

(A20) $$\begin{array}{c} {\mu^{\prime }}_{j}^{2}=\sum_{i=1}^{n}\alpha_{i}^{⋆}\mu_{i}^{\prime }\int \frac{f\left(x\right)p_{i}\left(x\right)p_{j}\left(x\right)}{{\left(\sum_{k=1}^{n}\alpha_{k}^{⋆}p_{k}\left(x\right)\right)}^{2}}dx. \end{array}$$

□

## Appendix D. An Alternative Perspective Based on the χ 2 Divergence

Let us define $\psi_{\alpha }=\sum_{k=1}^{n}\alpha_{k}p_{k}\left(x\right)$ and $\psi_{\beta }=\sum_{k=1}^{n}\beta p_{k}\left(x\right)$. Further, let us define $Z_{\frac{f\beta }{\alpha }}=\psi_{\beta }\left[\frac{f(x)}{\psi_{\alpha (x)}}\right]$. We also recall that the $\chi^{2}$ divergence between the pdf $\tilde{f}\left(x\right)=\frac{f(x)}{\mu }$ and ψ, is given by

(A21) $$\begin{array}{cc} \chi^{2}\left(\tilde{f},\psi \right) & =\int \frac{{\left(\tilde{f}\left(x\right)-\psi \left(x\right)\right)}^{2}}{\psi (x)}dx. \end{array}$$

Since in the randomized generalized balance heuristic estimator, G, all samples are simulated i.i.d. from $\alpha_{\beta }$, the variance can be expressed as

(A22)Vψβ[G]=1N2∑i=1nαi2βi2∑j=1niVψβ[f(Xi,j)ψα(Xi,j)](A23)=1N∑i=1nαi2βiVψβ[f(X)ψα(X)](A24)=1N∑i=1nαi2βiEψβ[f(X)ψα(X)−Zfβα2](A25)=1N∑i=1nαi2βi∫f(x)ψα(x)−Zfβα2ψβ(x)dx(A26)=1N∑i=1nαi2βi∫f(x)−Zfβαψα(x)2ψα(x)ψβ(x)ψα(x)dx(A27)=1N∑i=1nαi2βi∫μf˜(x)−Zfβαψα(x)2ψα(x)ψβ(x)ψα(x)dx,

where the integral can be seen as a modified $\chi^{2}$ divergence with two differences: (a) the normalizing constant $Z_{\frac{f\beta }{\alpha }}$ is modified with regard to μ when $\psi_{\alpha }\neq \psi_{\beta }$, and (b) there is the ratio $\frac{\psi_{\beta }}{\psi_{\alpha }}$ that appears multiplying (evoking an importance weight between the denominator we have used, $\psi_{\alpha }$, and the proposal used to simulate, $\psi_{\beta }$). Note that, when $\psi_{\alpha }=\psi_{\beta }$, $Z_{\frac{f\beta }{\alpha }}=\mu$, the ratio of mixtures is equal to one, and $V_{\psi_{\beta }}\left[G\right]=\frac{1}{N}\mu^{2}\chi^{2}\left(f,\psi_{\beta }\right)$.
